# Prevalence, Characteristics and Clonal Distribution of Extended-Spectrum β-Lactamase- and AmpC β-Lactamase-Producing *Escherichia coli* Following the Swine Production Stages, and Potential Risks to Humans

**DOI:** 10.3389/fmicb.2021.710747

**Published:** 2021-07-21

**Authors:** Soomin Lee, Jae-Uk An, Jae-Ho Guk, Hyokeun Song, Saehah Yi, Woo-Hyun Kim, Seongbeom Cho

**Affiliations:** College of Veterinary Medicine and Research Institute for Veterinary Science, Seoul National University, Seoul, South Korea

**Keywords:** extended-spectrum β-lactamase, AmpC β-lactamase, *Escherichia coli*, multidrug resistance, extra-intestinal pathogenic *E. coli*, virulence factor, clonal distribution, swine production stages

## Abstract

The worldwide spread of extended spectrum β-lactamase (ESBL)- and AmpC β-lactamase (AmpC)-producing *Escherichia coli* poses serious threats to public health. Swine farms have been regarded as important reservoirs of ESBL/AmpC-EC. This study aimed to determine the prevalence, ESBL/AmpC types, and clonal distribution of ESBL/AmpC-EC from swine farms and analyze the difference according to the swine production stages. In addition, we evaluated the potential risks of swine ESBL/AmpC-EC clones to humans. Individual fecal samples (*n* = 292) were collected from weaning, growing, finishing, and pregnant pigs in nine swine farms of South Korea between July 2017 and March 2020. In total, 161 ESBL/AmpC-EC isolates were identified (55.1%), with the highest prevalence detected in the weaning stage (86.3%). The dominant ESBL and AmpC types were CTX-M-55 (69.6%) and CMY-2 (4.3%), respectively. CTX-M found in all production stages, while CMY was only found in growing and finishing stages. In the conjugation assay, the high transferability of CTX-M gene (55.8%) was identified, while the transfer of CMY gene was not identified. The major clonal complexes (CCs) were CC101-B1 (26.8%), CC10-A (8.7%), and CC648-F (2.9%). There was similarity in clonal distribution between different swine production stages within swine farms, estimated using the *k*-means analysis, which suggested a clonal transmission between the different swine stages. Among swine ESBL/AmpC-EC sequence types (STs), seven STs (ST101, ST10, ST648, ST457, ST410, ST617, and ST744) were common with the human ESBL/AmpC-EC, which registered in National Center for Biotechnology Information database. The clonal population structure analysis based on the virulence factor (VF) presented that swine ESBL/AmpC-EC clones, especially ST101-B1, harbored a highly virulent profile. In conclusion, ESBL/AmpC-EC was distributed throughout the swine production stages, with the highest prevalence in the weaning stage. The CTX-M was present in all stages, while CMY was mostly found in growing-finishing stages. The swine ESBL/AmpC-EC was identified to harbor shared clone types with human ESBL/AmpC-EC and a virulent profile posing potential risk to humans. Considering the possibility of genetic and clonal distribution of ESBL/AmpC-EC among swine production stages, this study suggests the need for strategies considering the production system to control the prevalence of ESBL/AmpC-EC in swine farms.

## Introduction

The third-generation cephalosporin (3GC)-resistant Enterobacteriaceae, including the extended spectrum β-lactamase (ESBL)- or AmpC β-lactamase (AmpC)-producing *Escherichia coli* (ESBL/AmpC-EC), and the carbapenem Enterobacteriaceae, including carbapenemase (CP)-producing *E. coli* (CP-EC), have been reported as a serious global threat to public health. The 3GCs and carbapenems show excellent activity against Gram-positive and Gram-negative bacteria and are particularly prescribed in treating multidrug-resistant bacterial infections (Muller et al., 2018). The spread of ESBL/AmpC-EC or CP-EC is of great concern because it could aid the emergence and spread of pathogens that are difficult to treat even with an antimicrobial agent of choice regarded as a final treatment option (Nathisuwan et al., 2001; Cortes et al., 2010). In addition, as ESBL/AmpC- or CP-producing extra-intestinal pathogenic *E. coli* (ExPEC) clones are increasingly reported worldwide, the virulence potential of ESBL/AmpC-EC or CP-EC has also become an issue (Schaufler et al., 2016; Day et al., 2019; Santos et al., 2020).

Intensive use and misuse of β-lactam antibiotics, including penicillins, carbapenems, monobactams, and cephalosporins, in veterinary medicine has led to the emergence and spread ESBL/AmpC-EC in the animal husbandry (Dohmen et al., 2017). The increasing prevalence of ESBL/AmpC-EC in food-animal farms has been reported in multiple continents including Europe (Cortes et al., 2010; Schmithausen et al., 2015; Lupo et al., 2018), America (Cyoia et al., 2018; Moffat et al., 2020), Africa (Belmahdi et al., 2016; Alonso et al., 2017; Jouini et al., 2021), Australia (Abraham et al., 2015; Sparham et al., 2017), and Asia (Liu et al., 2018; Tansawai et al., 2019). In particular, pigs have been regarded as the main driver of the increasing prevalence of ESBL/AmpC-EC in food-animals (Bergspica et al., 2020). This epidemiologic trend may be attributed to the long-term and extensive usage of 3GC during swine production periods (Song et al., 2009; Peirano et al., 2010; Yoo et al., 2010; Barguigua et al., 2013; Hong et al., 2020). The possibility of ESBL-EC transmission from swine farms to humans has been continuously proposed and vice versa (Bok et al., 2020; Song et al., 2020). Various ESBL/AmpC-EC transmission routes have been suggested, including the food-chain of pigs (Liu et al., 2019), direct contacts of farm workers with pigs (Schmithausen et al., 2015), and manure excretion into the surrounding environment in farms such as soils, ponds, and rivers (Furlan and Stehling, 2018).

Swine production consists of four stages, including farrowing (birth to 3–4 weeks of age), weaning (4–7 weeks old), growing (7–14 weeks), and finishing stage (14–24 weeks old), and the farrow-to-finish swine farms refers to farms rearing all four swine production stages Pigs at each different stage in the farrow-to-finish swine farms are usually reared in three separated farrowing, weaning, and growing-finishing barns, respectively. However, there is a generally high probability of bacterial co-transmission between the production stages within a farm (Verhegghe et al., 2013; Fromm et al., 2014), through various routes, including farm workers and veterinarians, instrument contamination, or manure excretions into barns of different stages (Fromm et al., 2014; Schmithausen et al., 2015). Previous studies have reported the identification of ESBL/AmpC-EC throughout the swine production stages, with varying prevalence at each production stage (Schmithausen et al., 2015; Dohmen et al., 2017). Although not studied well until now, the characteristics and distribution of ESBL/AmpC-EC may also differ depending on the stage of swine production, and understanding their difference could be a cornerstone in reducing the prevalence of ESBL/AmpC-EC in swine farms.

The present study aimed to determine the prevalence, multidrug resistance (MDR), and virulence potential of ESBL/AmpC from swine farms. In addition, differences in β-lactamase types, antimicrobial susceptibility, and clonal distribution were analyzed according to the swine production stages. Finally, potential risks of swine ESBL/AmpC-EC clones on human health were evaluated.

## Materials and Methods

### Sample Collection

Samples were collected from nine farrow-to-finish swine farms located in four provinces in South Korea (four in Gyeonggi-do, three in Jeolla-do, one in Chungcheong-do, and one in Gyeongsang-do) between July 2017 and March 2020. Each farm was visited once (two farms in 2017, five farms in 2018, one farm in 2019, and one farm in 2020). Individual fecal samples were collected in similar numbers from each swine farm; a total of 26–34 pigs from each swine farm, including 5–6 weaning piglets (4–7 weeks old), 9–11 growing pigs (7–14 weeks old), 8–11 finishing pigs (14–24 weeks old), and 3–6 pregnant sows. A total of 292 samples from 51 weaning piglets, 96 growing pigs, 47 finishing pigs, and 50 pregnant sows were collected and transported immediately to a laboratory. All pigs included in this study were healthy without diarrhea.

### Isolation of ESBL/AmpC-EC and Non-ESBL/AmpC-EC

To isolate ESBL/AmpC-EC, 1 g of each fecal sample was homogenized with 9 ml of *E. coli* broth (Oxoid, United Kingdom) for 1 min using a homogenizer and incubated overnight at 37°C. Approximately 100 μl of enriched *E. coli* culture suspension was spread on MacConkey agar (Oxoid, United Kingdom). Cefotaxime (CTX) disk (30 μg/ml, Oxoid, United Kingdom) was then placed on the plate. After overnight incubation at 37°C, 2–4 3GC-resistant *E. coli* (3GC-EC) candidate isolates grown inside the CTX resistant zone (<22 mm) were selected and streaked on CHROMagar™ ESBL (CHROMagar, France) to demonstrate the morphology of 3GC-EC colony. One 3GC-EC isolate from each sample was randomly selected if more than one 3GC-EC isolates were identified from one sample. Finally, a standard double-disk test was performed to confirm the typical ESBL and AmpC phenotype, as described in the 2016–Clinical Laboratory Standard Institute (CLSI) guideline M100S 26th Edition.

To isolate non-ESBL/AmpC-EC, 10 μl of enriched *E. coli* broth suspension was streaked on MacConkey agar. Three colonies showing typical *E. coli* morphology were randomly selected and transferred to Eosin Methylene Blue (EMB) agar (Oxoid, United Kingdom) for purification. The suspected *E. coli* isolates on EMB agar were subjected to a standard ESBL/AmpC double-disk test, and typical non-ESBL/AmpC-EC isolates were determined as non-ESBL/AmpC-EC. One non-ESBL/AmpC-EC isolate per sample was randomly selected, where more than one non-ESBL/AmpC-EC isolates were identified from one sample. Considering the distribution of ESBL/AmpC-EC isolates by farm and production stage, a total of 81 non-ESBL/AmpC-EC isolates were selected for further analysis.

The presence of ESBL genes (*bla*_CTX-M_, *bla*_TEM_, and *bla*_SHV_), AmpC genes (*bla*_CMY_), and carbapenemase genes (*bla*_KPC_, *bla*_NDM_, and *bla*_OXA_) among ESBL/AmpC-EC isolates were determined with PCR. PCR amplicons were sequenced using the ABI PRISM 3730XL DNA analyzer (Applied Biosystems, United States). DNA sequences were compared with the published β-lactamase gene sequences available from the GenBank database of the NCBI using the BLAST program.^1^ Primer sequences and reaction conditions for each PCR-based genotyping of ESBL/AmpC are summarized in Supplementary Table 1.

### Antimicrobial Susceptibility Test

Disk diffusion susceptibility test (Kirby-Bauer method) were conducted for 14 antibiotics: amoxicillin/clavulanic acid (AMC, 20/10 μg), ampicillin (AMP, 10 μg), cefotaxime (CTX, 30 μg), ceftazidime (CAZ, 30 μg), ceftriaxone (CRO, 30 μg), aztreonam (ATM, 30 μg), imipenem (IMP, 10 μg), chloramphenicol (C, 30 μg), amikacin (AK, 30 μg), gentamicin (CN, 10 μg), tetracycline (TE, 30 μg), nalidixic acid (NA, 30 μg), ciprofloxacin (CIP, 5 μg), and trimethoprim–sulfamethoxazole (SXT, 1.25/23.75 μg). *Escherichia coli* ATCC 25922 was used as the reference strain for quality control. Antimicrobial susceptibility was interpreted according to the CLSI guidelines.

### Plasmid-Mediated Antimicrobial Resistance Genes, Intestinal Pathogenic *E. coli* Typing, and Extraintestinal Pathogenic *E. coli* Associated Virulence Factor Genotyping

The presence of plasmid-mediated antimicrobial resistance genes inferring resistance to chloramphenicol (*catA*, *cmlA*, and *floR*), tetracycline (*tetA*, *tetB*, and *tetD*), quinolone (*qnrA*, *qnrB*, *qnrC*, *qnrS*, and *aac(6)-Ib-cr*), aminoglycoside (*aac(3)-I*, *aac(3)-II*, and *aac(3)-IV*), and Sulfonamide/Trimethoprim (*dfrIa*, *dfrIb*, *dfrII*, *dfrVII*, and *dfrXII*) were determined using PCR. Intestinal pathogenic *E. coli* typing was conducted for the following types; shiga toxin-producing *E. coli* (*stx1* and *stx2*), enteropathogenic *E. coli* (*eaeA* and *bfpV*), enteroaggregative *E. coli* (*aggR*), enteroinvasive *E. coli* (*ipaH*), and enterotoxigenic *E. coli* (*lt*, *sta*, *stb*, and *east-1*). The extraintestinal pathogenic *E. coli* associated virulence factors (ExPEC VFs) associated with adhesion (*fimH*, *iha*, *papC*, and *csgA*), toxin (*astA*, *hlyA*, *aat*, *tsh*, and *pic*), protectin/serum resistance (*traT* and *ompT*), and siderophore (*fyuA* and *iroNe.coli*) were also determined using PCR. Primer sequences and reaction conditions are summarized in Supplementary Table 1.

### Biofilm Assay

Biofilm production assays were performed following a previously described protocol with modification (Naves et al., 2008). Briefly, overnight Luria-Bertani (LB) broth culture was diluted in fresh LB broth to a McFarland scale of 0.5. Approximately 120 μl of this dilution was added into 96-well microtiter plate and incubated for 24 h at 30°C in a stationary condition. Each bacterial suspension was inoculated into three wells of the microtiter plate. Growth optical densities (ODs) were measured at *λ* = 595 nm with a multiplate reader (Bio-rad, United States). The wells were then washed once with 200 μl of phosphate-buffered saline (PBS) dried for 20 min, and stained with 120 μl of 1% crystal violet for 5 min. This was followed by gentle washing with 200 μl of distilled water (DW) for four times and air-drying for 1 h. The absorbed dye was solubilized in 120 μl of absolute ethanol, and ODs were read at 595 nm. The extent of biofilm formation was calculated using the formula: $\mathrm{SBF}=\frac{\left(AB-CW\right)}{G}$, where SBF is the specific biofilm formation index, AB is the OD_595_ of the stained bacteria, CW is the OD_595_ of the stained control wells containing absolute media without bacteria, and G is the OD_595_ corresponding to cell growth in the media. An SBF value above 0.5 was suggested as positive biofilm formation. *E. coli* ATCC 25922 was used as the positive control, while the culture medium was used as the negative control.

### Expression of Biofilm-Associated Extracellular Matrix Components: Curli Fimbriae and Cellulose

To determine the expression of biofilm-associated extracellular matrix components (cellulose and curli fimbriae), a macrocolony assay was performed following a previously described protocol with modification (Schaufler et al., 2016). Approximately, 5 μl of an overnight culture from a single colony grown in 2 ml of LB medium was dropped on YESCA agar [10 g/L casamino acids (BD Bioscience, United States), 1 g/L yeast extract (BD Bioscience, United States), and 20 g/L agar (BD Bioscience, United States)] with Congo red solution [0.5% Congo red (Sigma-Aldrich, Germany) and 0.25% Coomassie brilliant blue (Sigma-Aldrich, Germany) diluted in ethanol]. Plates were incubated for 5 days at 28°C, and results were interpreted using the four morphotypes: rdar (red, dry, and rough; curli and cellulose), pdar (pink, dry, and rough; cellulose only), bdar (brown, dry, and rough; curli only), and saw (smooth and white; neither curli nor cellulose). *E. coli* ATCC 25922 was used as the negative control for the expression of curli fimbriae and cellulose.

### Plasmid Typing and Conjugation Assay

The replicon typing of ESBL/AmpC-EC was analyzed with the major plasmid incompatibility groups among Enterobacteriaceae (HI1, HI2, I1-Iγ, I2, X1, X2, X3, X4, L/M, FIA, FIB, FIC, FIIs, A/C, P, K B/O, and N) using a PCR-based replicon-typing method (Carattoli et al., 2005; Johnson et al., 2012; Lv et al., 2013). Conjugation assay was conducted with *E. coli* J53-Azi^R^ as the recipient and ESBL/AmpC-EC as the donors. LB agar plates containing 4 mg/L of CTX and 100 mg/L of sodium azide were used to select the transconjugants. The presence of the ESBL/AmpC genes (*bla_CTX-M_* and *bla_CMY_*), plasmid-mediated antimicrobial resistance genes, and replicon types in the transconjugants was confirmed by PCR. Primer sequences and reaction conditions are summarized in Supplementary Table 1.

### Clonal Distribution Analysis of Swine ESBL/AmpC-EC

#### Multi-Locus Sequence Typing and *E. coli* Phylogenetic Group Typing

Of the 161 ESBL/AmpC-EC isolates from the present study, 138 isolates were selected based on their antibiotic resistance and ExPEC VFs to analyze multi-locus sequence typing (MLST) and *E. coli* phylogenetic group typing. The MLST of ESBL/AmpC-EC isolates was performed as described previously (Wirth et al., 2006). A detailed scheme of gene amplification, allelic type, and sequence type (ST) assignment methods is available on the pubMLST website.^2^ The minimum spanning tree (MST) based on allelic profiles of seven MLST housekeeping genes was constructed using the BioNumerics software, version 6.6 (APPLIED MATHS, Belgium).

Seven *E. coli* phylogenetic groups (A, B1, B2, D, C, E, and F) were determined following a previously described protocol with application (Clermont et al., 2013). Primer sequences and reaction conditions for each PCR-based phylogenetic group analysis are summarized in Supplementary Table 1.

#### Similarity Analysis of the Clonal Distribution of Swine ESBL/AmpC-EC Following the Swine Production Stages Within Farms

We analyzed the similarity in the distribution of ST and phylogenetic groups following the swine production stages in each farm using the *k*-means similarity clustering algorithm based on Euclidean distance (Hartigan and Wong, 1979). To find the optimal number of clusters (*k*), we applied the average silhouette method (Lengyel and Botta-Dukat, 2019). The average silhouette method presumes that the optimal number of clusters *k* is the one that maximizes the average silhouette over a range of possible values for *k*. In this study, the optimal value of *k* was nine (Supplementary Figure 2). The *k*-means clustering analysis and the average silhouette method were conducted using the R software, version 4.3.2 (R foundation, Austria).

Based on the combination of nine swine farms (“farm A” to “farm I”) and four production stages (weaning piglets to sows), a total of 36 points indicating ST composition at each farm’s swine production stages were generated. Of the 36 points, three were excluded because there were no isolated ESBL/AmpC-EC strains from “growing stage” of “farm H,” “pregnant stage” of “farm H,” and “growing stage” of “farm I.” We clustered 33 points into nine clusters using the *k*-means algorithm based on the Euclidean distance of paired two points. The *k*-means clustering plot was generated using the R software, version 4.3.2 (R foundation, Austria).

### Analysis of the Potential Risk of Swine Farm-Derived ESBL/AmpC-EC Clones

#### Identification of the Shared Major STs of ESBL/AmpC-EC Isolated From Swine Farms and Humans

To identify the shared major STs between swine and human ESBL/AmpC-EC isolates, the whole genome sequence (WGS) data of human ESBL/AmpC-EC isolates registered in the NCBI Pathogen Isolates Browser^3^ were used.

WGS data of 11,269 human ESBL/AmpC-EC isolates from human hosts whose assembly data were available (accessed on 20 May 2021) were downloaded and analyzed to determine the MLST STs (Supplementary Material 1). In total, 739 STs were identified from 11,269 human ESBL/AmpC-EC isolates. We selected the major 20 STs of human ESBL/AmpC-EC isolates covering 73.8% of the total isolates (8,320/11,269) for further analysis.

#### Clonal Population Structure Analysis Based on the VFs and Phylogenetic Group Profiles

We investigated the potential risks of swine ESBL/AmpC-EC clones on humans by a clonal population structure analysis based on their ExPEC VFs and phylogenetic group profile using program STRUCTURE (Pritchard et al., 2000). Swine ESBL/AmpC-EC isolates were assigned into virulence profile populations (*k*) using a Baysian method in the program. The most likely number of populations (*k*) was defined by the value producing a maximal rate change in posterior probability, Δln (*k*; Evanno et al., 2005). The optimal number of populations *k* was five in the present study (Supplementary Figure 3). Assignment coefficients (Q values such as proportions of population *k*) were generated for each strain using the Markov chain Monte Carlo searches, which consisted of 100,000 burn-in steps followed by 100,000 iteration steps. Among the five populations, ESBL/AmpC-EC isolates were assigned to their best-fit populations based on the highest Q value. The Q values of individual ESBL/AmpC-EC are presented in a 100% stacked bar chart sorted by the STs.

### Statistical Analyses

The comparative analyses between groups (one stage vs. other stages, ESBL/AmpC-EC vs. non-ESBL/AmpC-EC) were performed using the generalized estimating equation (GEE) to adjust the farm-induced factors. In the GEE analysis, we presumed that several characteristics of the isolates could be affected by farm factors; therefore, we set the farm as “subject variable” and the number of isolates in each farm as “within subject variables.” Where zeros caused problems in calculating the odds ratio (OR) in the GEE analysis, the Fisher’s exact test was conducted by adding 0.5 to each cell (Pagano and Gauvreau, 2018). In the analysis of the differences in the average number of VFs and resistance to antibiotic classes between ESBL/AmpC-EC and non-ESBL/AmpC-EC, the student’s *t*-test was applied. All statistical analyses were conducted using the Statistical Package for the Social Sciences (SPSS) program, version 27.0 (IBM SPSS Statistics for Windows, United States).

## Results

### Prevalence, MDR, and Virulence Potential of ESBL/AmpC-EC in Swine Farms

#### Prevalence of ESBL/AmpC-EC in Swine Farms

**Figure 1:**
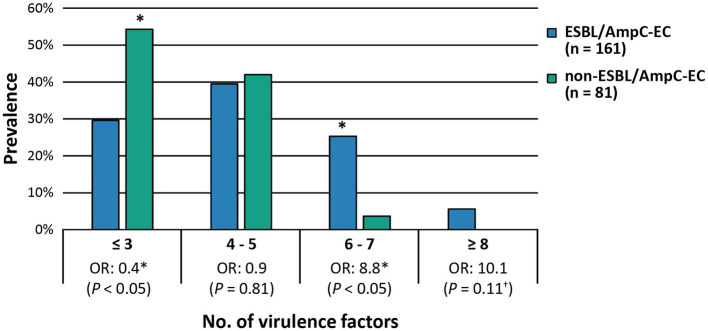
Prevalence of extended spectrum β-lactamase (ESBL)- or AmpC β-lactamase (AmpC)-producing *E. coli* (ESBL/AmpC-EC) according to different swine farms **(A)** and swine production stages **(B)**.

In total, 161 (55.1%) ESBL/AmpC-EC isolates were identified from 292 individual swine fecal samples. The farm prevalence of ESBL/AmpC-EC ranged from 17.6 to 89.7%, depending on the swine from which the samples were obtained (Figure 1A). Compared with other stages, weaning piglets (86.3%, 44/51) displayed the significantly highest prevalence of ESBL/AmpC-EC (*p* < 0.05), followed by growing pigs (58.3%, 51/96), finishing pigs (48.4%, 45/95), and pregnant sows (43.1%, 21/50; Figure 1B).

#### Higher MDR of ESBL/AmpC-EC Relative to Non-ESBL/AmpC-EC

**Figure 2:**
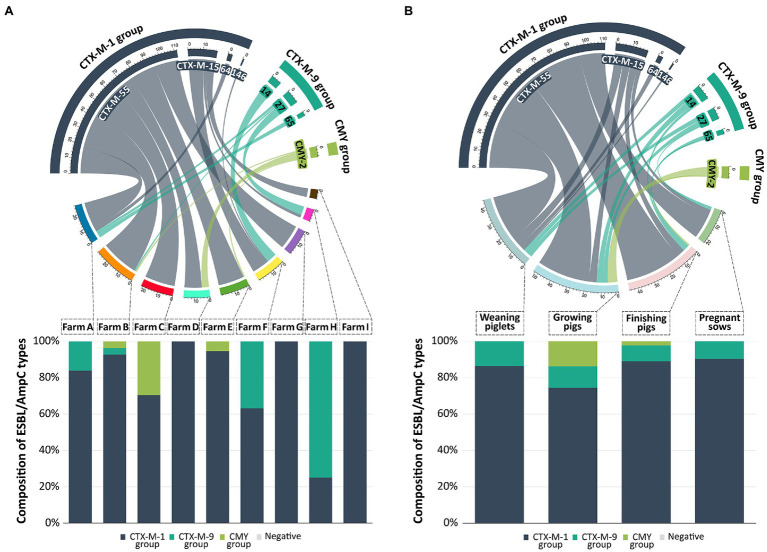
ESBL/AmpC-EC showed significantly higher resistance rate to antibiotics compared to non-ESBL/AmpC-EC. Statistically significant (^*^p < 0.05, GEE; ^†^*p* < 0.05, Chi-square test).

We investigated the antimicrobial resistance of the 161 ESBL/AmpC-EC isolates and compared them with those of the 81 non-ESBL/AmpC-EC isolates (Figure 2). Notably, ESBL/AmpC-EC isolates showed resistance to a higher number of antimicrobial classes (average: 5.7 antimicrobial classes) compared with that of non-ESBL/AmpC-EC isolates (average: 2.9 antimicrobial classes; *p* < 0.05). Furthermore, the MDR rate was significantly higher in the ESBL/AmpC-EC isolates (100%) compared with that in the non-ESBL/AmpC-EC isolates (63.0%; OR: 80.0, 95% CI: 11.68–547.88). Resistance to six antibiotics (AMC, CTX, CAZ, CRO, ATM, and AK) was found only in ESBL/AmpC-EC isolates but not in non-ESBL/AmpC-EC isolates. Except for the tetracycline class and carbapenem class, ESBL/AmpC producers showed a higher resistance rate for all antibiotic classes compared with that in non-ESBL/AmpC producers. Resistance to three of these antibiotic classes, which included broad-spectrum penicillin (OR: 100.8, 95% CI: 5.99–1694.62, *p* < 0.05), aminoglycoside (OR: 5.6, 95% CI: 2.08–15.26, *p* < 0.05), and quinolone (OR: 6.6, 95% CI: 3.31–12.99, *p* < 0.05), was significantly higher in the ESBL/AmpC-EC strains compared to non-ESBL/AmpC-EC.

In the antimicrobial resistance genotyping, the aminoglycoside resistance gene, *aac(3)-II*, was significantly more prevalent in the ESBL/AmpC-EC isolates (OR: 6.2, 95% CI: 1.25–30.70, *p* < 0.05) than in the non-ESBL/AmpC-EC (Supplementary Table 2).

#### ESBL/AmpC-EC With Multiple ExPEC VFs

All ESBL/AmpC-EC and non-ESBL/AmpC-EC were identified as non-pathogenic commensal *E. coli*, except for two ESBL/AmpC-EC isolates. One ESBL/AmpC-EC isolate was identified as enteropathogenic *E. coli* carrying heat stable ensterotoxin STa, and the other ESBL/AmpC-EC isolate was identified as atypical enteropathogenic *E. coli* carrying *eaeA* but not *bfpV*.

**Figure 3:**
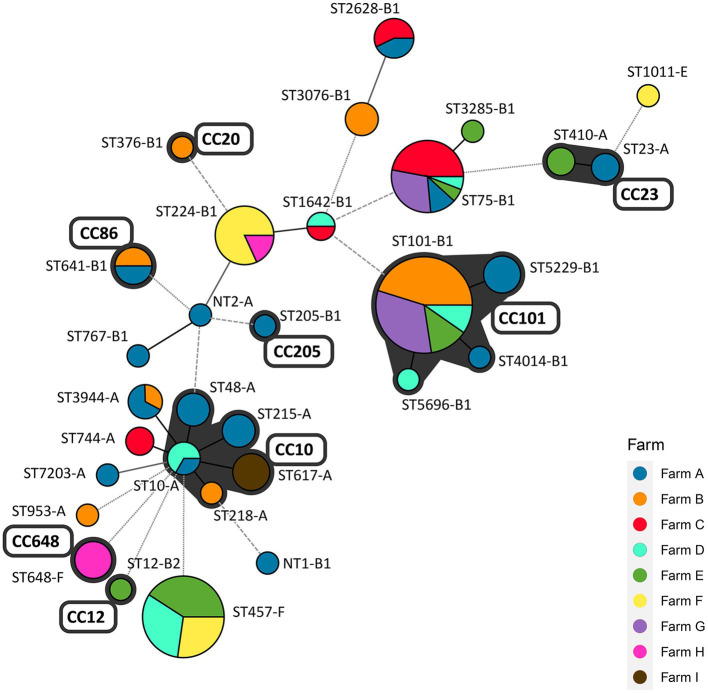
ESBL/AmpC-EC carried higher number of extra-intestinal pathogenic *E. coli* associated virulence factors (ExPEC VFs) compared to non-ESBL/AmpC-EC. OR, odds ratio. Statistically significant (^*^p < 0.05, GEEs; ^†^*p* < 0.05, Chi-square test).

A significantly higher number of ExPEC VFs were identified in the ESBL/AmpC-EC isolates (average: 4.6 VFs) compared with that in the non-ESBL/AmpC-EC isolates (average: 3.6 VFs; *p* < 0.05). The OR of having 6–7 VFs were 8.8-fold greater (95% CI: 1.31–59.30, *p* < 0.05) in the ESBL/AmpC-EC isolates than in the non-ESBL/AmpC-EC isolates (Figure 3). In contrast, the ESBL/AmpC-EC isolates showed a 0.4-fold less OR to harbor three or less VFs relative to that in the non-ESBL/AmpC-EC isolates (95% CI: 0.13–0.97, *p* < 0.05). Three VFs were highly prevalent in the ESBL/AmpC-EC isolates, namely pyelonephritis-associate pilus C, *papC* (OR: 19.8, 95% CI: 2.54–153.45, *p* < 0.05), serine protease pic autotransporter, *pic* (OR: 19.6, 95% CI: 1.16–330.30, *p* < 0.05), and outer membrane protease T, *ompT* (OR: 1.9, 95% CI: 1.00–3.64, *p* < 0.05), compared with the non-ESBL/AmpC-EC isolates (Table 1).

**Table 1:** Comparison of the carriage of extra-intestinal pathogenic *E. coli* associated virulence factors (ExPEC VFs) between extended spectrum β-lactamase (ESBL)- or AmpC β-lactamase (AmpC)-producing *E. coli* (ESBL/AmpC-EC) and non-ESBL/AmpC-EC isolates.

Virulence factor function	Virulence factor	ESBL/AmpC-EC (%) (*n* = 161)	Non-ESBL/AmpC-EC (%) (*n* = 81)	OR (95% CI)	*p*
Adhesion	*fimH*	96.3	96.3	1.0 (0.10–10.55)	1.00
	*iha*	3.1	0.0	5.8 (0.31–105.49)	0.24^†^
	*papC*	33.3	2.4	19.8 (2.54–153.45)	<0.01^*^
	*csgA*	99.4	95.1	8.4 (0.72–97.63)	0.09
Toxin	*hlyA*	4.3	6.1	0.7 (0.17–2.75)	0.60
	*astA*	11.7	19.5	0.4 (0.12–1.04)	0.06
	*aat*	0.6	3.7	0.2 (0.02–1.60)	0.11^†^
	*pic*	10.5	0.0	19.6 (1.16–330.30)	0.04^†^
	*tsh*	9.3	12.2	0.7 (0.18–2.94)	0.65
Siderophore	*fyuA*	28.4	11.0	3.2 (0.57–17.78)	0.19
	*iroNe.coli*	27.2	7.3	4.7 (0.70–31.13)	0.11
Protectin/Serum resistance	*ompT*	57.4	42.7	1.9 (1.00–3.64)	0.049^*^
	*traT*	77.2	63.4	2.0 (0.69–5.74)	0.20

CI, confidence interval. Statistically significant (

* *p* < 0.05, GEEs;

† *p* < 0.05, Chi-square test).

#### Improved Biofilm Formation of ESBL/AmpC-EC

The biofilm formation rate was higher in ESBL/AmpC-EC isolates (42.2%, 68/161) than that in non-ESBL/AmpC-EC isolates (16.0%, 13/81). The OR of biofilm formation was 3.8-fold greater in the ESBL/AmpC-EC isolates than in the non-ESBL/AmpC-EC isolates (95% CI: 1.42–10.30, *p* < 0.05). However, no significant differences were observed in the formation of two biofilm-associated extracellular matrix components, curli fimbriae (OR: 1.80, 95% CI: 0.71–4.55, *p* = 0.21) and cellulose (OR: 0.45, 95% CI: 0.10–2.07, *p* = 0.30), from the ESBL/AmpC-EC isolates relative to the non-ESBL/AmpC-EC isolates.

### Difference in the Distribution of β-Lactamases and Antimicrobial Susceptibility of ESBL/AmpC-EC According to the Swine Production Stages

#### Distribution of β-Lactamases in ESBL/AmpC-EC According to the Swine Production Stages

**Figure 4:**
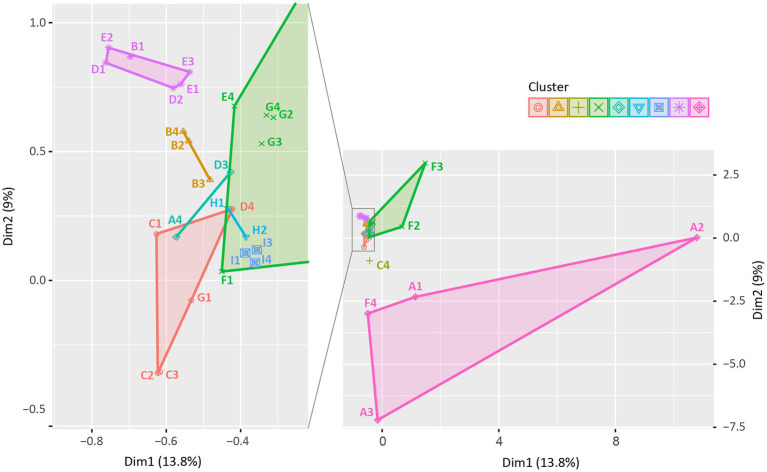
Prevalence and distribution of ESBL/AmpC types from swine farms. In the chord diagram, the size of segments on the top represent the number of ESBL/AmpC-EC isolates with a specific ESBL/AmpC types. Size of segments on the bottom represent the number of ESBL/AmpC-EC isolates detected in different farms **(A)** and production stages **(B)**. Ribbons connecting the top and bottom segments represent the number of ESBL/AmpC-EC isolates with a specific ESBL/AmpC type found on the respective farms and production stages. The connected bar chart shows the composition of ESBL/AmpC types based on the number of ESBL/AmpC-EC isolates in different farms **(A)** or production stages **(B)**. The chord diagram and bar chart were generated with R software (ver. 4.3.2).

Out of the 161 ESBL/AmpC-EC isolates, 154 isolates (95.7%, 154/161) were identified as ESBL-EC carrying the CTX-M family β-lactamases (CTX-M), while seven isolates (4.3%, 7/161) were identified as AmpC-EC carrying the CMY family β-lactamases (CMY; Figure 4). None of the isolates carried both CTX-M and CMY together. The CTX-M group was found in ESBL/AmpC-EC in all production stages and occupied 88.2–100.0% of the ESBL/AmpC-EC (Figure 4B). However, the CMY group was identified in ESBL/AmpC-EC isolates only in the growing (11.8%, 6/51) and finishing stages (2.2%, 1/45).

The CTX-M-1 group β-lactamase (83.9%, 135/161) was the most prevalent CTX-M; CTX-M-55 β-lactamase (69.6%, 112/161), CTX-M-15 (11.8%, 19/161), CTX-M-64 (1.9%, 3/161), and CTX-M-146 (0.6%, 1/161) belonged to this group (Figure 4). The second most prevalent CTX-M was the CTX-M-9 group β-lactamase (11.8%, 19/161); CTX-M-14 (5.6%, 9/161), CTX-M-27 (4.3%, 7/161), and CTX-M-65 (1.9%, 3/161) belonged to this group. All seven CMY-producing *E. coli* isolates was identified to carry CMY-2 β-lactamase. All ESBL/AmpC-EC carried only one type of CTX-M β-lactamase or CMY β-lactamase, and there were no isolates carrying more than one CTX-M or CMY β-lactamase. TEM β-lactamase was found in the 31 ESBL/AmpC-EC isolates (24.8%); however, all of these TEM β-lactamases were identified as TEM-1, which was a non-ESBL type in the sequencing analysis. Other ESBL types (CTX-M-2 group, CTX-M-8 group, CTX-M-25 group, and SHV) and carbapenemases (NDM, OXA, and KPC) were not identified in this study.

#### Antimicrobial Susceptibility of ESBL/AmpC-EC According to the Swine Production Stages

We compared the resistance rate of ESBL/AmpC-EC against 10 antimicrobial classes at specific stages to those of the other stages (Table 2). There were no significant differences in the resistance rate against all antimicrobial classes between the swine production stages, with the exception of β-lactamase inhibitor class. The resistance rate for the β-lactamase inhibitor class, which consisted of AMC, was significantly higher in growing pigs, compared with that of the other stages (OR: 9.8, 95% CI: 1.14–84.70, *p* < 0.05).

**Table 2:** Resistance rate of ESBL/AmpC-EC against antimicrobial classes following the swine production stages.

Anti-microbial classes	Anti-microbials	Weaning pigs			Growing pigs			Finishing pigs			Pregnant pigs		
		Prevalence (%)	OR (95% CI)	*p*	Prevalence (%)	OR (95% CI)	*p*	Prevalence (%)	OR (95% CI)	*p*	Prevalence (%)	OR (95% CI)	*p*
Pe	AMP	100.0	-	-	100.0	-	-	100.0	-	-	100	-	-
3rd Cepha	CTX, CAZ, CRO	100.0	-	-	100.0	-	-	100.0	-	-	100	-	-
Carba	IMP	0.0	-	-	0.0		-	0.0	-	-	0.0	-	-
Mono	ATM	45.5	1.0 (0.47–2.21)	0.96	44.2	1.0 (0.42–2.18)	0.92	48.9	1.2 (0.69–2.21)	0.47	38.1	0.7 (0.27–1.95)	0.52
Bi	AMC	0.0	0.1 (0.01–2.02)	0.14^†^	15.4	9.8 (1.14–84.70)	0.04^*^	4.4	0.6 (0.09–4.65)	0.65	0.0	0.6 (0.13–3.11)	0.73^†^
Phe	C	88.6	1.7 (0.53–5.42)	0.38	86.5	1.3 (0.62–2.93)	0.46	77.8	0.6 (0.25–1.25)	0.16	81.0	0.8 (0.48–1.27)	0.33
Ami	AK, CN	40.9	1.9 (0.84–4.13)	0.13	28.9	0.9 (0.35–2.17)	0.76	24.4	0.7 (0.28–1.51)	0.31	28.6	0.9 (0.27–2.93)	0.84
Te	TE	70.5	1.1 (0.67–1.64)	0.84	69.2	1.0 (0.39–2.37)	0.93	66.7	0.8 (0.47–1.44)	0.49	76.2	1.5 (0.56–3.79)	0.45
Qui	NA, CIP	81.8	1.8 (0.65–4.68)	0.27	76.9	1.2 (0.54–2.63)	0.66	68.9	0.7 (0.27–1.65)	0.38	66.7	0.6 (0.17–2.32)	0.49
S/T	SXT	52.3	0.8 (0.36–1.57)	0.45	65.4	1.6 (0.61–4.37)	0.33	60.0	1.2 (0.48–2.78)	0.74	42.9	0.5 (0.15–1.69)	0.27

The odds ratio (OR) was calculated on the antimicrobial resistance rate of ESBL/AmpC-EC from specific stage against those from other stages. OR, odds ratio; CI, confidence interval; Bi, β-lactamase inhibitor class; Pe, broad spectrum penicillin class; 3rd Cepha, 3rd cephalosporin class; Mono, monobactam class; Carba, carbapenem class; Phe, phenicol class; Ami, aminoglycoside class; Te, tetracycline class; Qui, quinolone class; S/T, sulfonamide/trimethoprim class; AMC, amoxicillin/clavulanate; AMP, ampicillin; CTX, cefotaxime; CAZ, ceftazidime; CRO, ceftriaxone; ATM, Aztreonam; IMP, imipenem; C, chloramphenicol; AK, amikacin; CN, gentamycin; TE, tetracycline; NA, nalidixic acid; CIP, ciprofloxacin; and SXT, sulfamethoxazole/trimethoprim.

Statistically significant (

* *p* < 0.05, GEEs;

† *p* < 0.05, Chi-square test).

#### Transferability of ESBL/AmpC Genes

To evaluate the horizontal transferability of ESBL/AmpC genes, we conducted the conjugation assay on 131 CTX-M and seven CMY β-lactamase-producing isolates (Supplementary Figure 1). The transferability of the CTX-M gene, *bla_CTX-M_*, was 58.8% (77/131); however, the transfer of the CMY gene *bla_CMY−2_* was not identified from all seven CMY-producing isolates. The most prevalent replicon type in the transconjugant of CTX-M was IncFIB (90.9%, 70/77), followed by IncI1-Iγ (28%, 17/77), IncI2 (6.5%, 5/78), and IncX4 (3.9%, 3/77). Various antibiotic resistance genes were transferred with *bla_CTX-M_*. The highest transferability of antimicrobial resistance gene was identified in *floR* (94.9%, 56/59), followed by *aac(3)-II* (85.7%, 6/7), and *qnrS1* (75.0%, 12/16).

### Clonal Distribution Analysis of ESBL/AmpC-EC From Swine Farms

#### Clonal Distribution of ESBL/AmpC-EC Between Swine Farms

**Figure 5:**
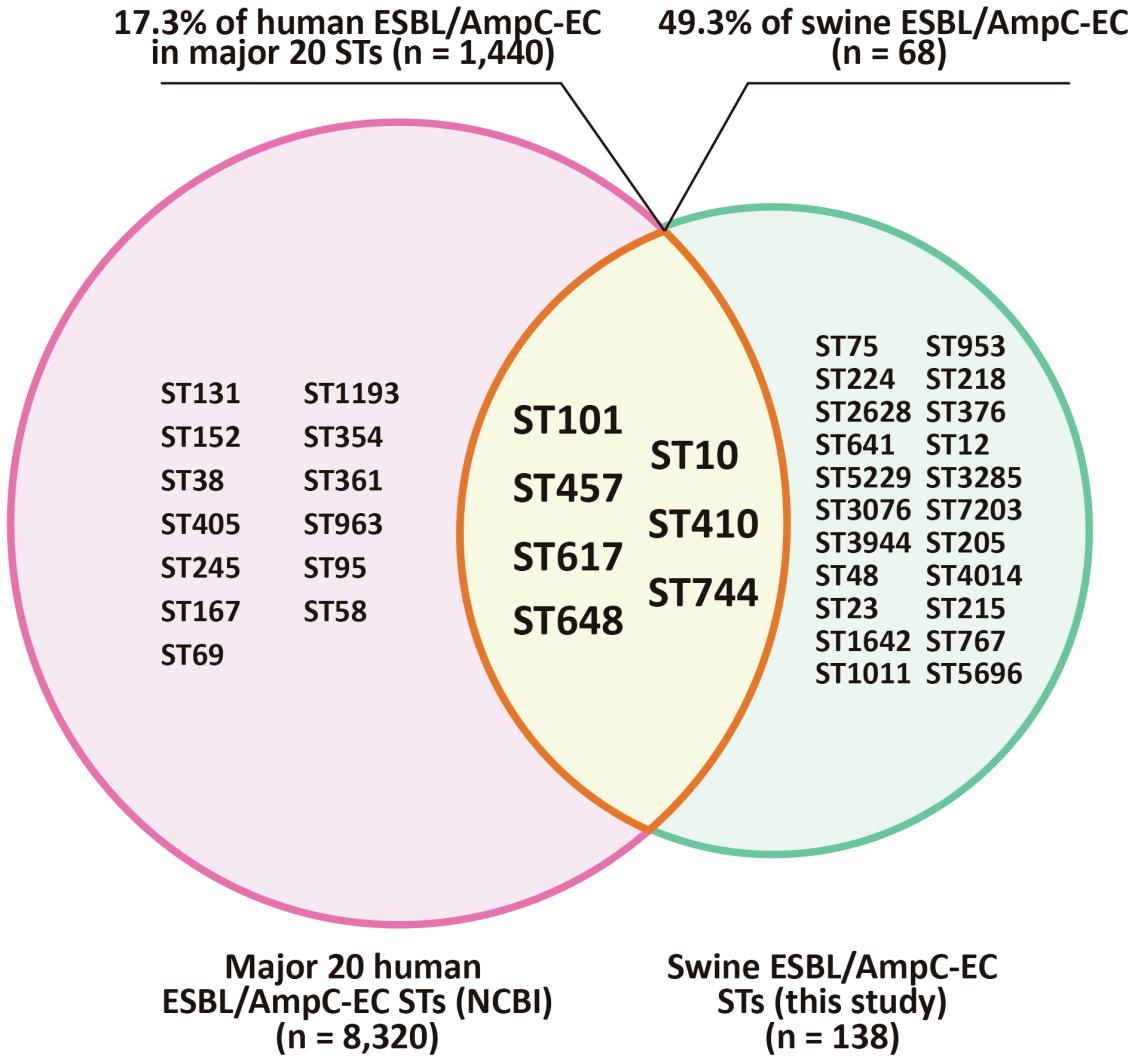
Minimum spanning tree (MST) based on allele profiles of multi-locus sequence type (MLST): clonal distribution of ESBL/AmpC-EC between pig farms. The number shows the sequence type of each node, and the size of the node indicates the number of strains belonging to the sequence type (ST)-phylogenetic group. The gray shadow represents the clonal complex (CC). Branch line types represent differences in the number of alleles: bold solid line (1 allele), thin solid line (2–3 alleles), dashed line (4 alleles), and dotted line (above 5 alleles). CC, clonal complex; ST, sequence type; NT, non-typable ST (including two different non-typable STs).

The MLST and the seven *E. coli* phylogenetic group (A, B1, B2, D, C, E, and F) typing analysis were conducted to analyze the clonal distribution of the swine farm-derived ESBL/AmpC-EC isolates (Figure 5). In the MLST analysis, a total of eight CCs were identified, and CC101-B1 (26.8%, 37/138), CC10-A (8.7%, 13/138), CC648-F (2.9%, 4/138), and CC23-A (2.9%, 4/138) were the major CCs. Around 31 STs, including two non-typable STs, were identified, with ST101-B1 (22.8%, 31/138), ST457-F (16.2%, 22/138), ST75-B1 (12.5%, 17/138), and ST224-B1 (8.1%, 11/138) as the major STs.

A total of 40.6% (56/138) of the ESBL/AmpC-EC isolates belonged to four CCs (CC101-B1, CC10-A, CC86-B1, and CC23-A), which were shared across two and more swine farms. A total of 73.5% (100/138) of ESBL/AmpC-EC isolates belonged to nine STs (ST10-A, ST101-B1, ST457-F, ST75-B1, ST224-A, ST641-B1, ST3944-A, ST-2628-B1, and ST1642-B1), which were shared across two and more swine farms.

#### Similarity of Clonal Distribution Among Swine Production Stages Within Farms

**Figure 6:**
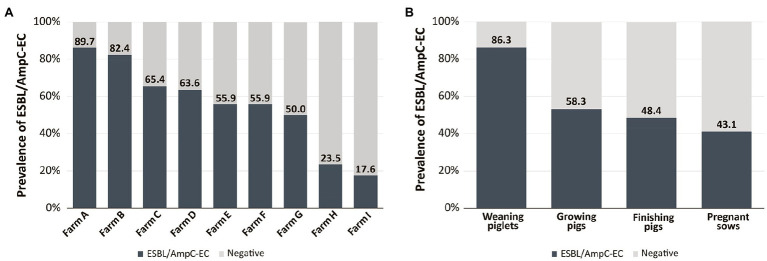
*k*-means similarity clustering plot: similarity in the clonal distribution among swine production stages within farms. A total of 33 points were described and clustered into 9 (symbols and colors) using the *k*-means similarity clustering algorithm based on Euclidean distance. The *k*-means cluster plot was generated using the R software (ver. 4.3.2). Each point indicates the distribution of STs among swine production stages in each farm, consisting of 36 points based on combination of 9 farms (A to I) and 4 production stages (1, weaning piglets; 2, growing pigs; 3, finishing pigs; and 4, pregnant sows; e.g., A1 presents the clonal distribution of “Weaning piglets” of “Farm A”). Three points (H2, H4, and I2) were excluded as no ESBL/AmpC-EC strains were isolated from “Growing pigs” of “Farm H”, “Pregnant sows” of “Farm H,” and “Growing pigs” of “Farm I.”

We evaluated the similarity in the clonal distribution of different production stages within the farms using the *k*-means similarity clustering algorithm (Figure 6; Supplementary Table 3). Distributions of STs and phylogenetic groups for each farm and production stage combination were presented in Supplementary Table 3. In Figure 6, 33 points represent the clonal distribution following the combination of nine farms (“farm A” to “farm I”) and four stages (“weaning piglets” to “pregnant sows”). In the *k*-means clustering analysis, the points were clustered into nine clusters based on the similarity distance between them, and 2–7 points belonged to each cluster. We found that the clonal distribution of three or all stages in the same farm was clustered together, thereby showing similarity, except for “farm D.” The clonal distribution of two stages in “farm D” were clustered together.

### Analysis of the Potential Risk of Swine Farm-Derived ESBL/AmpC-EC Clones

#### Identification of the Shared Major STs of ESBL/AmpC-EC Isolated From Swine Farms and Humans

**Figure 7:**
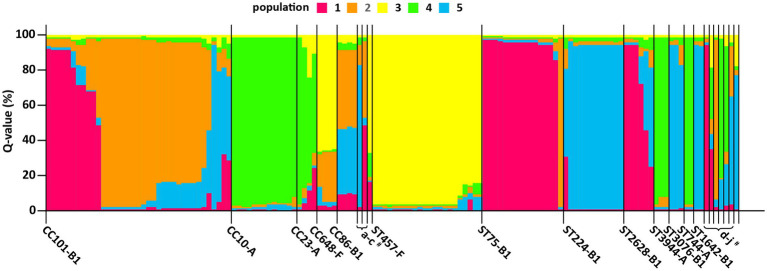
Shared major STs of ESBL/AmpC-EC isolated from swine farms and humans. Venn diagram shows the STs shared between swine farm derived ESBL/AmpC-ECs from this study and ESBL/AmpC-ECs from human sources which registered in the NCBI Pathogen Isolation Database. The intersection area of the two circles represents to the seven shared STs (ST101, ST10, ST457, ST410, ST617, ST744, and ST648) of ESBL/AmpC-EC from swine farms and human sources. ST, sequence type.

To identify the shared major STs of ESBL/AmpC-EC isolates in swine farms and human sources, we compared the STs of the swine ESBL/AmpC-EC isolates with the major 20 STs of the human ESBL/AmpC-EC isolates, registered in the NCBI Pathogen Isolates Browser database (Figure 7).

Among 29 MLST STs from the swine ESBL/AmpC-EC isolates, seven STs (ST101, ST10, ST457, ST410, ST617, ST744, and ST648) were shared with the human ESBL/AmpC-EC strains. These seven STs contained 68 (49.3%) of the 138 ESBL/AmpC-EC isolates in the swine farms and 1,440 (17.3%) of the 8,320 ESBL/AmpC-EC strains belonging to the major 20 STs from human sources.

#### Clonal Population Structure Analysis of Swine ESBL/AmpC-EC Based on ExPEC VFs and Phylogenetic Group Profiles

**Figure 8:**
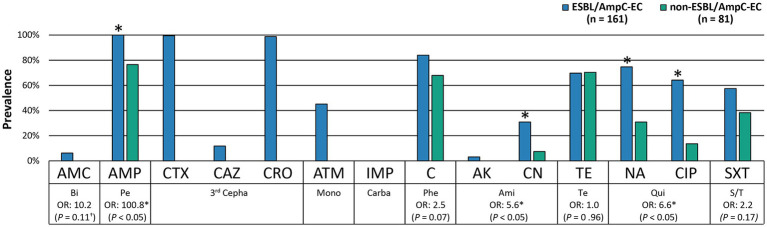
A clonal population analysis of swine ESBL/AmpC-EC isolates using program structure. Each isolate was assigned to five populations based on their ExPEC VFs and *E. coli* phylogenetic group profile. The ExPEC VFs and *E. coli* phylogenetic group profile for each clonal population are presented in Table 2. Each isolate is represented by a vertical segment and aligned horizontally according to CCs and STs (x-axis). The proportion of population (Q value) for each isolate is shown as 100% stacked bar plots, with proportions of colored sections representing the probability of belonging to each population within each segment (y-axis). CC, clonal complex; ST, sequence type; STNT, ST non-typable. ^#^a, CC205-B1; b, CC376-B1; c, CC12-B2; d, ST3285-B1; e, ST953-A; f, NT-B1; g, ST7203-A; h, STNT-A; I, ST767-B1; and j, ST1011-E.

We conducted a clonal population structure analysis based on ExPEC VFs and phylogenetic group profiles of ESBL/AmpC-EC using the program STRUCTURE to evaluate the potential risk of swine ESBL/AmpC-EC clones to humans. In this clonal population analysis, 138 ESBL/AmpC-EC isolates were divided into five populations, and each population contained 24–34 ESBL/AmpC-EC isolates. Individual ESBL/AmpC-EC isolates assigned to the populations were visualized as a 100% stacked bar chart sorted by the STs (Figure 8). Differences in the ExPEC VFs and phylogenetic groups in each population are presented in Table 3. The clonal population distribution of swine ESBL/AmpC-EC isolates for each ST-phylogenetic group is presented in Supplementary Table 4.

**Table 3:** ExPEC VFs and phylogenetic group profiles for each clonal structure population.

Population	Phylogenetic group (n^a^)	Mean no. of VF^b^	Prevalence (%) of:												
			Adhesion				Toxin					Siderophore		Protectin/Serum resistance	
			*fimH*	*iha*	*papC*	*csgA*	*hlyA*	*astA*	*pic*	*tsh*	*aat*	*fyuA*	*iroN e.coli*	*ompT*	*traT*
1	B1 (33) A (1)	6.7	100.0	11.8	82.4	100.0	14.7	2.9	32.4	11.8	0.0	23.5	97.1	94.1	97.1
2	B1 (28)	4.8	100.0	0.0	0.0	100.0	0.0	0.0	0.0	39.3	0.0	78.6	0.0	60.7	100.0
3	*F* (26) B2 (1)	4.2	100.0	0.0	44.4	96.3	0.0	7.4	0.0	0.0	0.0	18.5	3.7	88.9	55.6
4	A (24)	3.0	83.3	0.0	8.3	100.0	0.0	4.2	0.0	0.0	0.0	16.7	0.0	12.5	75.0
5	B1 (24) E (1)	3.0	100.0	0.0	4.0	100.0	0.0	28.0	4.0	0.0	0.0	4.5	8.0	8.0	44.0

a Number of isolates.

b Extra-intestinal pathogenic *E. coli* associated virulence factors (ExPEC VFs).

The ESBL/AmpC-EC isolates in population 1 mainly identified as CC101-B1 and ST75-B1, and harbored the highest number (average 6.7) of VFs, which was characterized with the highest prevalence of *papC*, *hlyA*, *pic*, *iroNe.coli*, and *ompT*. Isolates in population 2 mainly identified as CC101-B1 and CC86-B1. This population carried the second highest number of VFs (average: 4.8 VFs), characterized by the high prevalence of *tsh* and *fyuA*. Isolates in population 3 mainly identified as CC648-F and ST457-F (average: 4.2 VFs), with a high prevalence of *papC* and *ompT*. Isolates in population 4 mainly identified as CC10-A and CC23-A (average: 3.0 VFs). Finally, isolates in population 5 identified as ST224-B1 (average: 3.0 VFs). Isolates in populations 4 and 5 carried the lowest number of VFs.

## Discussion

The emergence and spread of 3GC-EC, including ESBL/AmpC-EC, has been reported by the WHO as a worldwide public health concern (WHO, 2017). Swine farm husbandry has been regarded as an important reservoir of ESBL/AmpC-EC (Carattoli, 2008). In the present study, we identified that more than half of pigs harbored ESBL/AmpC-EC isolates (55.1%, 161/292), and the prevalence varied depending on the swine production stages. The weaning piglets showed the significantly highest prevalence compared to pigs in the other stages (86.3%, *p* < 0.05; Figure 1B). Consistently, the high prevalence of ESBL/AmpC-EC in the weaning stage has been described in previous studies in Denmark (Hansen et al., 2013), Netherlands (Dohmen et al., 2017), and Germany (Schmithausen et al., 2015). We presumed that the high usage of β-lactams during the weaning stage, compared to other stages, could be one of major causes of the high prevalence of ESBL/AmpC-EC at the weaning stage. According to studies that investigated the patterns of antibiotics use in a global swine production system, over 70% β-lactams used in the swine industry was applied between birth and 10 weeks of age (Sjolund et al., 2016; Burow et al., 2019; Lekagul et al., 2019; Korsgaard et al., 2020). β-lactams have been commonly prescribed for the treatment and prevention of postweaning syndromes, including postweaning diarrhea, edema disease, and endotoxin shock, which are the major problems in swine industry (van Beers-Schreurs et al., 1992). Considering that colonization of ESBL/AmpC-EC could last longer than 6 months even without antibiotic selection pressure, ESBL/AmpC-EC colonies in the intestinal tract of swine at the weaning stage could persist until the date of their slaughter (about 150–230 days old; Kennedy and Collignon, 2010). In that point, we suggest that ESBL/AmpC-EC prevalence at the weaning stage could be a reflection of that of the entire farm, and that weaning stage should serve as the critical point in controlling the prevalence of ESBL/AmpC-EC in swine farms.

Notably, ESBL/AmpC-EC from swine farms showed MDR, multiple virulence factors, and enhanced biofilm formation ability relative to non-ESBL/AmpC-EC. Swine farm-derived ESBL/AmpC-EC isolates had a 100% MDR rate and a higher resistance rate against antibiotics, including broad-spectrum penicillins, aminoglycosides, and quinolones, compared with those in the non-ESBL/AmpC-EC (*p* < 0.05). In addition, ExPEC VFs, which were involved in human ExPEC infections, such as urinary tract infections, sepsis, and meningitis, were also highly identified in ESBL/AmpC-EC than in the non-ESBL/AmpC-EC (*p* < 0.05). Especially, the prevalence of the three VFs, namely *papC*, *pic*, and *ompT*, which were reported to show a positive association with the high mortality in human ExPEC infection (Kudinha et al., 2013; Krawczyk et al., 2015; Dutra et al., 2020), was significantly higher in the ESBL/AmpC-EC isolates (*p* < 0.05). Furthermore, the OR of biofilm formation was 3.8-fold greater (95% CI: 1.42–10.30, *p* < 0.05) in the ESBL/AmpC-EC isolates than in the non-ESBL/AmpC-EC isolates. Biofilm formation conferred fitness advantage to the bacteria by enhancing their survivability, increasing their virulence, and facilitating their ability to acquire virulence and antibiotic resistance genes during horizontal gene transmission due to their high microbial density (Donlan and Costerton, 2002; Schroeder et al., 2017). Collectively, these enhanced properties, namely MDR, multiple virulence factors, and enhanced biofilm formation ability, of ESBL/AmpC-EC from swine farms could make them difficult to be controlled once introduced to swine farms, allowing ESBL/AmpC-EC to survive and continue to exist within swine farms. In addition, the ESBL/AmpC-EC, with these properties, in swine farms could be transmitted to humans through the chain of pig meat (Liu et al., 2019), direct contact with the farm workers (Schmithausen et al., 2015), and manure excretion into the farm’s surrounding environment, including soils, ponds, and rivers (Furlan and Stehling, 2018). Spread of ESBL/AmpC-EC strains can be of serious public health concern since it could aid in the emergence of pathogens, which are difficult to control in the food-animal industry as well as human hospitals (Yoo et al., 2010; Dantas Palmeira and Ferreira, 2020).

The CTX-M β-lactamase (CTX-M) and CMY β-lactamase (CMY) are the most predominant type of ESBLs and AmpCs in 3GC-EC isolates from humans and animals (Philippon et al., 2002; Canton et al., 2012). In this study, all ESBL/AmpC-EC isolates carried CTX-M (95.7%, 154/161) or CMY (4.3%, 7/161); however, their distribution differed according to the swine production stages. CTX-M was prevalent in all four swine production stages, while CMY was only identified from growing (85.7%, 6/7) and finishing stages (14.3%, 1/7). We presumed that higher use of the ceftiofur during the growing-finishing stages, compared to other stages, could be one reason for the higher prevalence of CMY at growing-finishing stages. According to the previous studies, ceftiofur has been mainly prescribed to treat swine respiratory infection diseases, and these diseases has been reported to show higher prevalence in growing-finishing stages, compared to other stages (Hornish and Katarski, 2002; Timmerman et al., 2006; Tantituvanont et al., 2009; Deng et al., 2015). In general, CTX-M and CMY had different resistance profile against ceftiofur, a member of 3GC; CMY usually carried resistance against ceftiofur, while CTX-M does not (Deng et al., 2015). CMY prevalence has been reported to be positively associated with the usage of ceftiofur in the swine industry (Hornish and Katarski, 2002; Timmerman et al., 2006; Deng et al., 2015). In addition to higher usage of ceftiofur during growing-finishing stages, we suggest that the low horizontal gene transferability of CMY genes (0.0%, 0/7) compared to CTX-M genes (59.5%, 78/131) identified from this study could also be another reason for the accumulation of CMY producers only at growing-finishing stages, without spreading into other stages. Our results suggest that 3GC-resistant strains with different characteristics could exist depending on the swine production stage, which proposes the need to consider the production stage in the studies of 3GC-resistant bacteria in swine farms.

In the present study, the most prevalent ESBL was CTX-M-55 (69.6%, 112/161), followed by CTX-M-15 (11.8%, 19/161) and CTX-M-14 (5.6%, 9/161). CTX-M-15 and CTX-M-14 are regarded as the most prevalent types of ESBLs worldwide since the last decade (Song et al., 2009; Kennedy and Collignon, 2010; Shin et al., 2017). CTX-M-15 and CTX-M-14 have been reported as still dominant ESBL types in Australia (Abraham et al., 2015; Sparham et al., 2017) and Africa (Belmahdi et al., 2016; Alonso et al., 2017). However, the prevalence of CTX-M-55 has been recently increasing in Europe (Rossolini et al., 2008; Haenni et al., 2018), North America (Moffat et al., 2020), and Asia (Lee et al., 2013; Zhou et al., 2018; Tansawai et al., 2019), suggesting the possibility of global epidemiological shift in ESBL types. In this study, all seven AmpC-producing isolates were identified to carry CMY-2 (4.3%, 7/161), which was the most frequent reported CMY type found in human- and food-animal-derived AmpC producers worldwide (Wu et al., 2018).

The horizontal gene transfer system plays a crucial role in the transmission of ESBL/AmpC-EC; however, the clonal transfer could also be important in their transmission (Dohmen et al., 2017). Schmithausen et al. (2015) reported that ESBL-EC from individual pigs could spread into farm environments and almost the entire pigs present within the compartment could be affected by ESBL-EC from individual pigs (Schmithausen et al., 2015). When the clonal distribution in the swine production stages was compared *via* the *k*-means clustering analysis, we found that the clonal distribution of three or all stages from the same farm tended to be clustered together, showing similarity of clonal distribution. A similar clonal distribution between production stages implied the presence of predominantly colonized ESBL/AmpC-EC clone types throughout the farm and the spread of these strains between production stages. Our result suggests that through the repeated cycle, which involved the shedding from swine through feces, survival in the farm environment, and reintroduction to swine, the ESBL/AmpC-EC could spread into other swine at different stages and could continue to exist within swine farms.

Here, the most prevalent CCs of ESBL/AmpC-EC from swine farms was found to be CC101-B1 (26.8%, 37/138), followed by CC10-A (8.7%, 13/138) and CC648-F (2.9%) in the molecular epidemiological analysis. Consistently, a study on the ESBL-EC isolates from swine at slaughterhouses in South Korea reported CC101 as the major CC type of ESBL-EC from pigs followed by CC10 (Song et al., 2020). In contrast, the most common CC of ESBL/AmpC-EC was CC10 in other countries including Portugal, Netherlands, Taiwan, and China, while the second most common CC varied across studies, including CC155, CC405, or CC648 (Ramos et al., 2013; Hammerum et al., 2014; Zhang et al., 2016; Lee and Yeh, 2017; Liu et al., 2018). In the comparison analysis of the major ESBL/AmpC clone types from swine farms and human sources, we identified that seven STs (ST101, ST10, ST648, ST457, ST410, ST617, and ST744) were shared between swine farm-derived ESBL/AmpC-EC and human ESBL/AmpC-EC. The ST10, a major ST of CC10, has been reported to be one of the most important pandemic human ESBL/AmpC-producing ExPEC clones (ST131, ST10, ST69, ST73, and ST95) since 2000s (Manges et al., 2019). The ST101, a major ST in CC101, has been reported worldwide, although not yet regarded as a member of the pandemic clones. ESBL/AmpC-EC clone type ST101 has attracted renewed global attention in human ESBL/AmpC-producing ExPEC infections given its enhanced virulence and pan-drug resistance (Yoo et al., 2013; Manges et al., 2019; Santos et al., 2020), and has been reported to cause hemolytic uremic syndrome (Mellmann et al., 2008) and bloodstream infections (Santos et al., 2020). Consistently, in the clonal population structure analysis based on the ExPEC VF profiles and phylogenetic groups using the program STRUCTURE, we also identified a highly virulent profile of ESBL/AmpC-EC ST101 clone. In this analysis, the population 1, showing a highly virulent profile carrying a higher number of VFs associated with adhesion, toxin, siderophores, and serum resistance, consisted mainly of ESBL/AmpC-EC clone type ST101 along with ST75 strains, compared to other clone type strains.

In conclusion, ESBL/AmpC-EC, carrying MDR and enhanced virulence potential, was distributed throughout the swine production stages with the highest prevalence at the weaning stage. CTX-M β-lactamase was the dominant ESBL type and was identified in all four swine production stages, while CMY β-lactamase was identified only in growing and finishing stages. The similarity in the clonal distribution between different swine production stages within farms was identified, which suggested a possibility of clonal transmission between the different swine stages. ESBL/AmpC-EC from swine farms was identified to harbor a virulent profile posing potential risk to humans and shared clone types with ESBL/AmpC-EC from human sources. To further explore the possibility of clonal transmission from swine farms to humans, additional comparative analysis studies based on the WGS of ESBL/AmpC-EC from swine farms and humans would need to be carried out. Our study results suggest the need for strategies considering the swine production system to control the prevalence of ESBL/AmpC-EC in swine farms.

## Data Availability Statement

The original contributions presented in the study are included in the article/Supplementary Material, further inquiries can be directed to the corresponding authors.

## Author Contributions

SC and W-HK conceived and designed the study. W-HK, J-UA, and SL analyzed the epidemiologic data. HS, SY, J-HG, J-UA, and SL performed the sampling and experiments. SC, W-HK, and SL prepared and reviewed the manuscript. SL was a major contributor, both in experiments and writing of the manuscript. All authors contributed to the article and approved the submitted version.

### Conflict of Interest

The authors declare that the research was conducted in the absence of any commercial or financial relationships that could be construed as a potential conflict of interest.

## Footnotes

^1^https://blast.ncbi.nlm.nih.gov/Blast.cgi

^2^https://pubmlst.org/

^3^https://www.ncbi.nlm.nih.gov/pathogens/isolates/
