# Supplementary material for: Prevalence, Characteristics and Clonal Distribution of Extended-Spectrum β-Lactamase- and AmpC β-Lactamase-Producing Escherichia coli Following the Swine Production Stages, and Potential Risks to Humans
Source: Front Microbiol. 2021 Jul 21;12:710747. doi: 10.3389/fmicb.2021.710747 (PMC8334370; doi:10.3389/fmicb.2021.710747)
Supplement: Supplementary file 1 [file Image_1.pdf]

frontiers

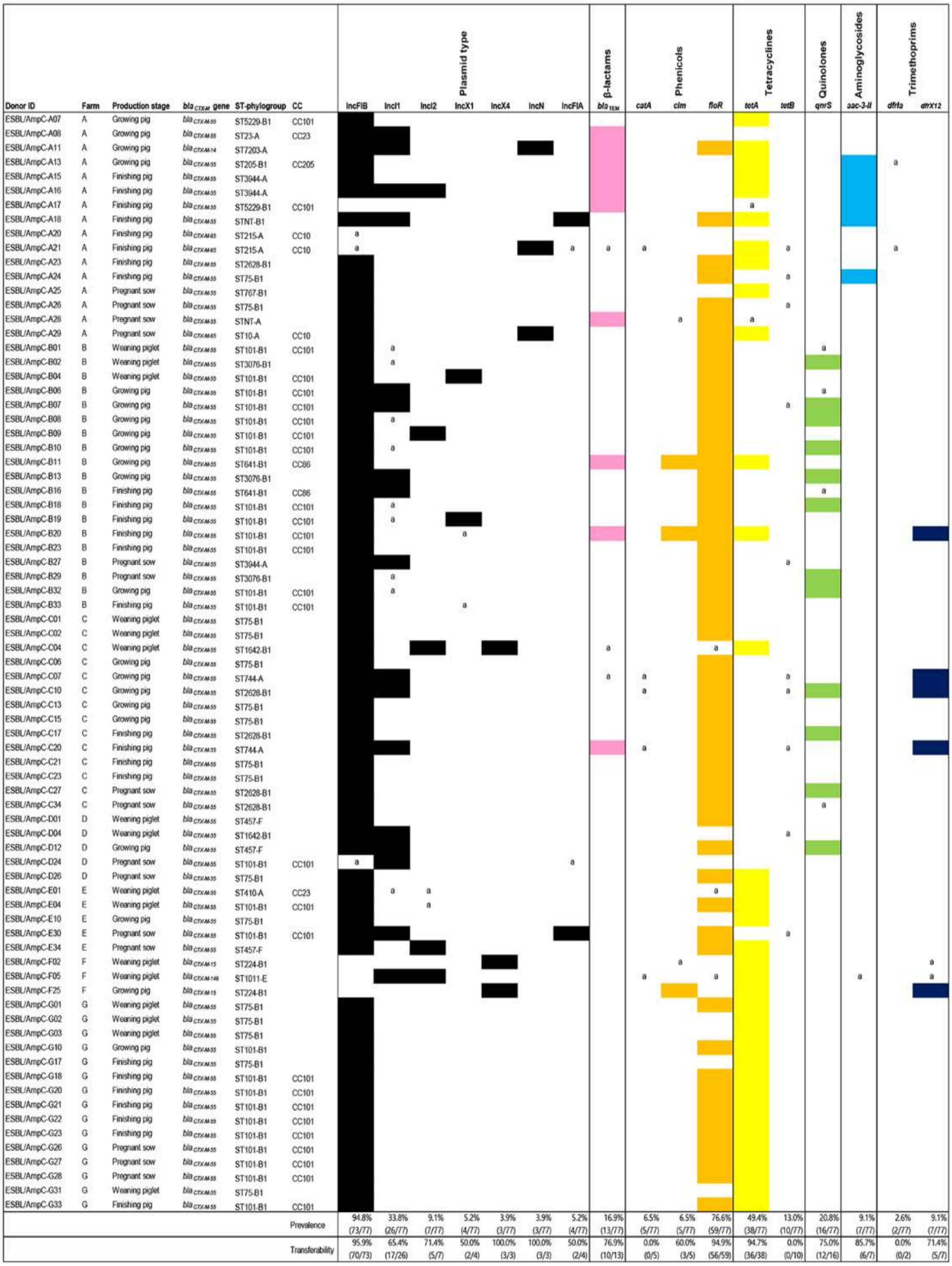

Plasmid type
β-lactams
Phenicols
Tetracyclines
Quinolones
Aminoglycosides
Trimethoprims
Donor ID
Farm
Production stage
bla CTX-M gene
ST-phylogroup
CC
IncFIB
IncI1
IncI2
IncX1
IncX4
IncN
IncFIA
bla TEM
catA
cml
floR
tetA
tetB
qnrS
aac-3-II
dfrla
dfrX12
ESBL/AmpC-A07 A Growing pig bla CTX-M-55 ST5229-B1 CC101
ESBL/AmpC-A08 A Growing pig bla CTX-M-55 ST23-A CC23
ESBL/AmpC-A11 A Growing pig bla CTX-M-14 ST7203-A
ESBL/AmpC-A13 A Growing pig bla CTX-M-55 ST205-B1 CC205
ESBL/AmpC-A15 A Finishing pig bla CTX-M-55 ST3944-A
ESBL/AmpC-A16 A Finishing pig bla CTX-M-55 ST3944-A
ESBL/AmpC-A17 A Finishing pig bla CTX-M-55 ST5229-B1 CC101
ESBL/AmpC-A18 A Finishing pig bla CTX-M-55 STNT-B1
ESBL/AmpC-A20 A Finishing pig bla CTX-M-65 ST215-A CC10
ESBL/AmpC-A21 A Finishing pig bla CTX-M-65 ST215-A CC10
ESBL/AmpC-A23 A Finishing pig bla CTX-M-55 ST2628-B1
ESBL/AmpC-A24 A Finishing pig bla CTX-M-55 ST75-B1
ESBL/AmpC-A25 A Pregnant sow bla CTX-M-55 ST767-B1
ESBL/AmpC-A26 A Pregnant sow bla CTX-M-55 ST75-B1
ESBL/AmpC-A28 A Pregnant sow bla CTX-M-55 STNT-A
ESBL/AmpC-A29 A Pregnant sow bla CTX-M-65 ST10-A CC10
ESBL/AmpC-B01 B Weaning piglet bla CTX-M-55 ST101-B1 CC101
ESBL/AmpC-B02 B Weaning piglet bla CTX-M-55 ST3076-B1
ESBL/AmpC-B04 B Weaning piglet bla CTX-M-55 ST101-B1 CC101
ESBL/AmpC-B06 B Growing pig bla CTX-M-55 ST101-B1 CC101
ESBL/AmpC-B07 B Growing pig bla CTX-M-55 ST101-B1 CC101
ESBL/AmpC-B08 B Growing pig bla CTX-M-55 ST101-B1 CC101
ESBL/AmpC-B09 B Growing pig bla CTX-M-55 ST101-B1 CC101
ESBL/AmpC-B10 B Growing pig bla CTX-M-55 ST101-B1 CC101
ESBL/AmpC-B11 B Growing pig bla CTX-M-55 ST641-B1 CC86
ESBL/AmpC-B13 B Growing pig bla CTX-M-55 ST3076-B1
ESBL/AmpC-B16 B Finishing pig bla CTX-M-55 ST641-B1 CC86
ESBL/AmpC-B18 B Finishing pig bla CTX-M-55 ST101-B1 CC101
ESBL/AmpC-B19 B Finishing pig bla CTX-M-55 ST101-B1 CC101
ESBL/AmpC-B20 B Finishing pig bla CTX-M-55 ST101-B1 CC101
ESBL/AmpC-B23 B Finishing pig bla CTX-M-55 ST101-B1 CC101
ESBL/AmpC-B27 B Pregnant sow bla CTX-M-55 ST3944-A
ESBL/AmpC-B29 B Pregnant sow bla CTX-M-55 ST3076-B1
ESBL/AmpC-B32 B Growing pig bla CTX-M-55 ST101-B1 CC101
ESBL/AmpC-B33 B Finishing pig bla CTX-M-55 ST101-B1 CC101
ESBL/AmpC-C01 C Weaning piglet bla CTX-M-55 ST75-B1
ESBL/AmpC-C02 C Weaning piglet bla CTX-M-55 ST75-B1
ESBL/AmpC-C04 C Weaning piglet bla CTX-M-55 ST1642-B1
ESBL/AmpC-C06 C Growing pig bla CTX-M-55 ST75-B1
ESBL/AmpC-C07 C Growing pig bla CTX-M-55 ST744-A
ESBL/AmpC-C10 C Growing pig bla CTX-M-55 ST2628-B1
ESBL/AmpC-C13 C Growing pig bla CTX-M-55 ST75-B1
ESBL/AmpC-C15 C Growing pig bla CTX-M-55 ST75-B1
ESBL/AmpC-C17 C Finishing pig bla CTX-M-55 ST2628-B1
ESBL/AmpC-C20 C Finishing pig bla CTX-M-55 ST744-A
ESBL/AmpC-C21 C Finishing pig bla CTX-M-55 ST75-B1
ESBL/AmpC-C23 C Finishing pig bla CTX-M-55 ST75-B1
ESBL/AmpC-C27 C Pregnant sow bla CTX-M-55 ST2628-B1
ESBL/AmpC-C34 C Pregnant sow bla CTX-M-55 ST2628-B1
ESBL/AmpC-D01 D Weaning piglet bla CTX-M-55 ST457-F
ESBL/AmpC-D04 D Weaning piglet bla CTX-M-55 ST1642-B1
ESBL/AmpC-D12 D Growing pig bla CTX-M-55 ST457-F
ESBL/AmpC-D24 D Pregnant sow bla CTX-M-55 ST101-B1 CC101
ESBL/AmpC-D26 D Pregnant sow bla CTX-M-55 ST75-B1
ESBL/AmpC-E01 E Weaning piglet bla CTX-M-55 ST410-A CC23
ESBL/AmpC-E04 E Weaning piglet bla CTX-M-55 ST101-B1 CC101
ESBL/AmpC-E10 E Growing pig bla CTX-M-55 ST75-B1
ESBL/AmpC-E30 E Pregnant sow bla CTX-M-55 ST101-B1 CC101
ESBL/AmpC-E34 E Pregnant sow bla CTX-M-55 ST457-F
ESBL/AmpC-F02 F Weaning piglet bla CTX-M-15 ST224-B1
ESBL/AmpC-F05 F Weaning piglet bla CTX-M-148 ST1011-E
ESBL/AmpC-F25 F Growing pig bla CTX-M-15 ST224-B1
ESBL/AmpC-G01 G Weaning piglet bla CTX-M-55 ST75-B1
ESBL/AmpC-G02 G Weaning piglet bla CTX-M-55 ST75-B1
ESBL/AmpC-G03 G Weaning piglet bla CTX-M-55 ST75-B1
ESBL/AmpC-G10 G Growing pig bla CTX-M-55 ST101-B1
ESBL/AmpC-G17 G Finishing pig bla CTX-M-55 ST75-B1
ESBL/AmpC-G18 G Finishing pig bla CTX-M-55 ST101-B1 CC101
ESBL/AmpC-G20 G Finishing pig bla CTX-M-55 ST101-B1 CC101
ESBL/AmpC-G21 G Finishing pig bla CTX-M-55 ST101-B1 CC101
ESBL/AmpC-G22 G Finishing pig bla CTX-M-55 ST101-B1 CC101
ESBL/AmpC-G23 G Finishing pig bla CTX-M-55 ST101-B1 CC101
ESBL/AmpC-G26 G Pregnant sow bla CTX-M-55 ST101-B1 CC101
ESBL/AmpC-G27 G Pregnant sow bla CTX-M-55 ST101-B1 CC101
ESBL/AmpC-G28 G Pregnant sow bla CTX-M-55 ST101-B1 CC101
ESBL/AmpC-G31 G Weaning piglet bla CTX-M-55 ST75-B1
ESBL/AmpC-G33 G Finishing pig bla CTX-M-55 ST101-B1 CC101
Prevalence 94.8% (73/77) 33.8% (26/77) 9.1% (7/77) 5.2% (4/77) 3.9% (3/77) 3.9% (3/77) 5.2% (4/77) 16.9% (13/77) 6.5% (5/77) 6.5% (5/77) 76.6% (59/77) 49.4% (38/77) 13.0% (10/77) 20.8% (16/77) 9.1% (7/77) 2.6% (2/77) 9.1% (7/77)
Transferability 95.9% (70/73) 65.4% (17/26) 71.4% (5/7) 50.0% (2/4) 100.0% (3/3) 100.0% (3/3) 50.0% (2/4) 76.9% (10/13) 0.0% (0/5) 60.0% (3/5) 94.9% (56/59) 94.7% (36/38) 0.0% (0/10) 75.0% (12/16) 85.7% (6/7) 0.0% (0/2) 71.4% (5/7)

Supplementary Figure 1. Transferability of the β-lactamase gene, resistance genes, and replicon types in ESBL/AmpC-EC in the conjugation assay. Colored cells indicate the transfer of genes/plasmids represented by each column in the conjugation assay with *Escherichia coli* J53-Azi^R strain. The transferability of genes was confirmed by PCR. [a] detected in donor strains but did not transfer; Blank = plasmid replicon or antimicrobial resistance genes were not detected in donor strains
