# Supplementary material for: Prevalence, Characteristics and Clonal Distribution of Extended-Spectrum β-Lactamase- and AmpC β-Lactamase-Producing Escherichia coli Following the Swine Production Stages, and Potential Risks to Humans
Source: Front Microbiol. 2021 Jul 21;12:710747. doi: 10.3389/fmicb.2021.710747 (PMC8334370; doi:10.3389/fmicb.2021.710747)
Supplement: Supplementary file 2 [file Image_2.pdf]

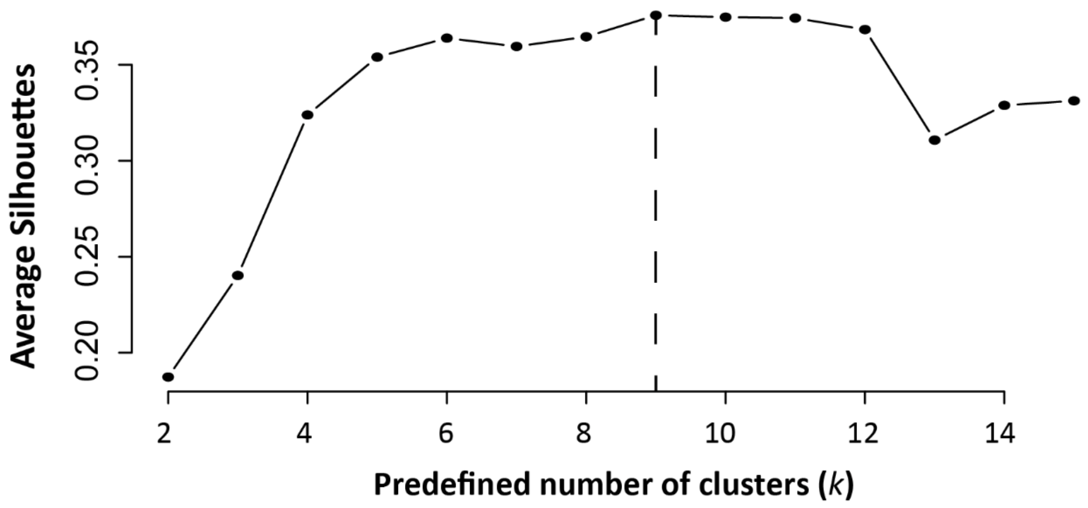

Supplementary Figure 2. Determination of the best-fit cluster number by average silhouettes method for the *k*-means clustering algorithm. An average silhouette method presumes that the optimal number of clusters *k* is the one that maximizes the average silhouette over a range of possible values for *k* in the *k*-means clustering algorithm.
