# Supplementary material for: Prevalence, Characteristics and Clonal Distribution of Extended-Spectrum β-Lactamase- and AmpC β-Lactamase-Producing Escherichia coli Following the Swine Production Stages, and Potential Risks to Humans
Source: Front Microbiol. 2021 Jul 21;12:710747. doi: 10.3389/fmicb.2021.710747 (PMC8334370; doi:10.3389/fmicb.2021.710747)
Supplement: Supplementary file 3 [file Image_3.pdf]

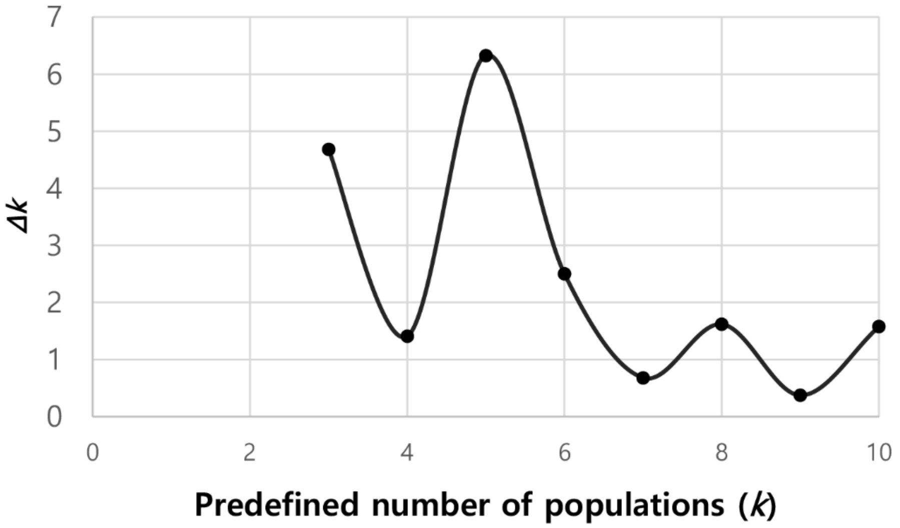

Supplementary Figure 3. Determination of the best-fit population number using the Bayesian approach for population structure analysis. Δ*k* is the second-order rate of change of *k*. A clear peak shows the most likely value of *k* (5) in the population structure analysis.
