# Supplementary material for: Prevalence, Characteristics and Clonal Distribution of Extended-Spectrum β-Lactamase- and AmpC β-Lactamase-Producing Escherichia coli Following the Swine Production Stages, and Potential Risks to Humans
Source: Front Microbiol. 2021 Jul 21;12:710747. doi: 10.3389/fmicb.2021.710747 (PMC8334370; doi:10.3389/fmicb.2021.710747)
Supplement: Supplementary file 4 [file Table_1.pdf]

Supplementary Table 1. Oligonucleotide primers and annealing temperature used in this study.

Function	Genes		Nucleotide sequence	Amplicon size (bp)	Annealing temp. (°C)	Reference
ESBL types	*bla*$_{CTX-M-1}$ group	F	GTTACAATGTGTGAGAAGCAG	1,041	60	(Jouini et al., 2007)
		R	CCGTTTCCGCTATTACAAAC			
	bla$_{CTX-M-2}$ group	F	CGACGCTACCCCTGCTATT	832	60	(Jouini et al., 2007)
		R	CAGAAACCGTGGGTTACGAT			
	bla$_{CTX-M-8}$ group	F	GGCGCTGGAGAAAAGCAG	862	60	(Jouini et al., 2007)
		R	GGTTTTATCCCCGACAACC			
	bla$_{CTX-M-9}$ group	F	GTGACAAAGAGAGTGCAACGG	857	60	(Jouini et al., 2007)
		R	ATGATTCTCGCCGCTGAAGCC			
	bla$_{CTX-M-25}$ group	F	GCACGATGACATTCGGG	327	60	(Jouini et al., 2007)
		R	AACCCACGATGTGGGTAGC			
	bla$_{CMY}$	F	AACACACTGATTGCGTCTGAC	1,226	60	(Jouini et al., 2007)
		R	CTGGGCCTCATCGTCAGTTA			
	bla$_{SHV}$	F	TCGCCTGTGTATTATCTCCC	768	54	(Jouini et al., 2007)
		R	CGCAGATAAATCACCACAATG			
	bla$_{TEM}$	F	TCCGCTCATGAGACAATAACC	1,057	58	(Jouini et al., 2007)
		R	ACGCTCAGTGGAACGAAAAC			
	bla$_{OXA}$	F	ACACAATACATATCAACTTCGC	813	60	(Jouini et al., 2007)
		R	AGTGTGTTTAGAATGGTGATC			
Phylogenetic group	*yjaA*	F	CAAACGTGAAGTGTCAGGAG	288	55	(Clermont et al., 2013)
		R	AATGCGTTCCTCAACCTGTG			
	chuA	F	ATGGTACCGGACGAACCAAC	211	55	(Clermont et al., 2013)
		R	TGCCGCCAGTACCAAAGACA			
	tspE4.C2	F	CACTATTCGTAAGGTCATCC	152	55	(Clermont et al., 2013)
		R	AGTTTATCGCTGCGGGTCGC			
	AceK.f	F	AACGCTATTCGCCAGCTTGC	400	55	(Clermont et al., 2013)
	ArpA1.r	R	TCTCCCCATACCGTACGCTA			
	ArpAgpE.f	F	GATTCCATCTTGTCAAAATATGCC	301	55	(Clermont et al., 2013)
	ArpAgpE.r	R	GAAAAGAAAAAGAATTCCCAAGAG			
	trpAgpC.1	F	AGTTTTATGCCCAGTGCGAG	219	55	(Clermont et al., 2013)
	trpAgpC.2	R	TCTGCGCCGGTCACGCCC			
	trpBA.f	F	CGGCGATAAAGACATCTTCAC	489	55	(Clermont et al., 2013)
	trpBA.r	R	GCAACGCGGCCTGGCGGAAG			
Virulence factors	*fimH*	F	TGCAGAACGGATAAGCCGTGG	508	63	(Gonzalez Moreno et al., 2020)
		R	GCAGTCACCTGCCCTCCGGTA			
	iha	F	CTGGCGGAGGCTCTGAGATCA	827	55	(Aazam et al., 2012)
		R	TCCTTAAGCTCCCGCGGCTGA			
	papC	F	GTGGCAGTATGAGTAATGACCGTTA	200	63	(Zhi et al., 2016)
		R	ATATCCTTTCTGCAGGGATGCAATA			
	csgA	F	ACTCTGACTTGACTATTACC	200	55	(Gonzalez Moreno et al., 2020)
		R	AGATGCAGTCTGGTCAAC			
	astA	F	TGCCATCAACACAGTATATCCG	102	65	(Muller et al., 2007)
		R	ACGGCTTTGTAGTCCTTCCAT			
	hlyA	F	AACAAGGATAAGCACTGTTCTGGCT	1,176	63	(Aazam et al., 2012)
		R	ACCATATAAGCGGTCATTCCCGTCA			
	aat	F	CTGGCGAAAGACTGTATCAT	629	53	(Moyo et al., 2007)
		R	CAATGTATAGAAATCCGCTGTT			
	tsh	F	ACTATTCTCTGCAGGAAGTC	824	55	(Aazam et al., 2012)
		R	CTTCCGATGTTCTGAACGT			
	pic	F	AGCCGTTTCCGCAGAAGCC	1,111	63	(Muller et al., 2007)
		R	AAATGTCAGTGAACCGACGATTGG			
	traT	F	GGTGTGGTGCGATGAGCACAG	290	60	(Gonzalez Moreno et al., 2020)
		R	CACGGTTCAGCCATCCCTGAG			
	ompT	F	ATCTAGCCGAAGAAGGAGGC	559	64	(Aazam et al., 2012)

		R	CCCGGGTCATAGTGTTCATC			
	fyuA	F	TGATTAACCCCGCGACGGGAA	880	63	(Gonzalez Moreno et al., 2020)
		R	CGCAGTAGGCACGATGTTGTA			
	iroNe.coli	F	AAGTCAAAGCAGGGGTTGCCCG	665	63	(Aazam et al., 2012)
		R	GACGCCGACATTAAGACGCAG			
Antimicrobial resistance	*catA*	F	AGTTGCTCAATGTACCTATAACC	547	57	(Saenz et al., 2004)
		R	TTGTAATTCATTAAGCATTCTGCC			
	cmlA	F	CCGCCACGGTGTTGTTGTTATC	698	57	(Saenz et al., 2004)
		R	CACCTTGCCTGCCCATCATTAG			
	floR	F	TATCTCCCTGTCGTTCCAG	399	52	(Saenz et al., 2004)
		R	AGAACTCGCCGATCAATG			
	tetA	F	GCTACATCCTGCTTGCCTTC	210	58	(Saenz et al., 2004)
		R	CATAGATCGCCGTGAAGAG			
	tetB	F	TTGGTTAGGGGCAAGTTTTG	659	56	(Saenz et al., 2004)
		R	GTAATGGGCCAATAACACCG			
	tetD	F	AAACCATTACGGCATTCTGC	787	60	(Saenz et al., 2004)
		R	GACCGGATACACCATCCATC			
	qnrA	F	ATTTCTCA CGCCAGGATTTG	516	53	(Liao et al., 2015)
		R	GATCGGCAAAGGTTAGGTCA			
	qnrB	F	GATCGTGAAAGCCAGAAAGG	469	53	(Liao et al., 2015)
		R	ACGATGCCTGGTAGTTGTCC			
	qnrC	F	GGGTTGTACATTTATTGAATC	447	50	(Liao et al., 2015)
		R	TCCACTTTACGAGGTTCT			
	qnrS	F	ACGACATTCGTCAACTGCAA	417	53	(Liao et al., 2015)
		R	TAAATTGGCACCCTGTAGGC			
	aac(6)-Ib-cr	F	TTGCGATGCTCTATGAGTGGCTA	482	50	(Liao et al., 2015)
		R	CTCGAATGCCTGGCGTGTTT			
	aac(3)-I	F	ACCTACTCCCAACATCAGCC	169	60	(Saenz et al., 2004)
		R	ATATAGATCTCACTACGCGC			
	aac(3)-II	F	ACTGTGATGGGATACGCGTC	237	60	(Saenz et al., 2004)
		R	CTCCGTCAGCGTTTCAGCTA			
	aac(3)-IV	F	CTTCAGGATGGCAAGTTGGT	286	60	(Saenz et al., 2004)
		R	TCATCTCGTTCTCCGCTCAT			
	dfrIa	F	GTGAAACTATCACTAATGG	474	55	(Saenz et al., 2004)
		R	TTAACCCTTTTGCCAGATTT			
	dfrIb	F	GAGCAGCTICTITTIAAAGC	393	60	(Saenz et al., 2004)
		R	TTAGCCCTTTIICCAATTTT			
	dfrII	F	GATCACGTGCGCAAGAAATC	141	50	(Saenz et al., 2004)
		R	AAGCGCAGCCACAGGATAAAT			
	dfrVII	F	TTGAAAATTTCATTGATT	474	55	(Saenz et al., 2004)
		R	TTAGCCTTTTTTCCAAATCT			
	dfrXII	F	GGTGSGCAGAAGATTTTTCGC	319	60	(Saenz et al., 2004)
		R	TGGGAAGAAGGCGTCACCCTC			
Replicon types	IncHI1	F	GGAGCGATGGATTACTTCAGTAC	471	60	(Carattoli et al., 2005)
		R	TGCCGTTTCACCTCGTGAGTA			
	IncHI2	F	TTTCTCCTGAGTCACCTGTTAACAC	644	60	(Carattoli et al., 2005)
		R	GGCTCACTACCGTTGTCATCCT			
	IncI1-Iγ	F	CGAAAGCCGGACGGCAGAA	139	60	(Carattoli et al., 2005)
		R	TCGTCGTTCCGCCAAGTTCGT			
	IncI2	F	CTGTCGGCATGTCTGTCTC	553	55	(Lv et al., 2013)
		R	CTGGCTACCAGTTGCTCTAA			
	IncX1	F	GCTTAGACTTTGTTTTATCGTT	461	62	(Johnson et al., 2012)
		R	TAATGATCCTCAGCATGTGAT			
	IncX2	F	GCGAAGAAATCAAAGAAGCTA	678	63	(Johnson et al., 2012)
		R	TGTTGAATGCCGTTCTTGTCCAG			
	IncX3	F	GTTTTCTCCACGCCCTTGTTCA	351	63	(Johnson et al., 2012)
		R	CTTTGTGCTTGGCTATCATAA			
	IncX4	F	AGCAAACAGGGAAAGGAGAAGACT	569	62	(Johnson et al., 2012)
		R	TACCCCAAATCGTAACCTG			
	IncL/M	F	GGATGAAAACTATCAGCATCTGAAG	785	60	(Carattoli et al., 2005)

	R	CTGCAGGGGCGATTCTTTAGG			
IncFIA	F	CCATGCTGGTTCTAGAGAAGGTG	462	60	(Carattoli et al., 2005)
	R	GTATATCCTTACTGGCTTCCGCAG			
IncFIB	F	GGAGTTCTGACACACGATTTTCTG	702	63	(Carattoli et al., 2005)
	R	CTCCCGTCGCTTCAGGGCATT			
IncFIC	F	GTGAACTGGCAGATGAGGAAGG	262	60	(Carattoli et al., 2005)
	R	TTCTCCTCGTCGCCAAACTAGAT			
IncFIIs	F	CTGTCGTAAGCTGATGGC	270	60	(Carattoli et al., 2005)
	R	CTCTGCCACAAACTTCAGC			
IncA/C	F	GAGAACCAAAGACAAAGACCTGGA	465	60	(Carattoli et al., 2005)
	R	ACGACAAACCTGAATTGCCTCCTT			
IncP	F	CTATGGCCCTGCAAACGCGCCAGAAA	534	60	(Carattoli et al., 2005)
	R	TCACGCGCCAGGGCGCAGCC			
IncK	F	GCGGTCCGGAAAGCCAGAAAAC	160	60	(Carattoli et al., 2005)
	R	TCTTTCACGAGCCCGCCAAA			
IncB/O	F	GCGGTCCGGAAAGCCAGAAAAC	159	60	(Carattoli et al., 2005)
	R	TCTGCGTTCCGCCAAGTTCGA			
IncN	F	GTCTAACGAGCTTACCGAAG	559	55	(Carattoli et al., 2005)
	R	GTTTCAACTCTGCCAAGTTC			

References

Aazam, K., Hassan, M., and Mahbobe, M. (2012). Detection of uropathogenic Escherichia coli virulence factors in patients with urinary tract infections in Iran. *African Journal of Microbiology Research* 6(39)**,** 6811-6816. doi: 10.5897/ajmr12.1462.

Carattoli, A., Bertini, A., Villa, L., Falbo, V., Hopkins, K.L., and Threlfall, E.J. (2005). Identification of plasmids by PCR-based replicon typing. *J Microbiol Methods* 63(3)**,** 219-228. doi: 10.1016/j.mimet.2005.03.018.

Clermont, O., Christenson, J.K., Denamur, E., and Gordon, D.M. (2013). The Clermont Escherichia coli phylo-typing method revisited: improvement of specificity and detection of new phylo-groups. *Environ Microbiol Rep* 5(1)**,** 58-65. doi: 10.1111/1758-2229.12019.

Gonzalez Moreno, C., Torres Luque, A., Oliszewski, R., Rosa, R.J., and Otero, M.C. (2020). Characterization of native Escherichia coli populations from bovine vagina of healthy heifers and cows with postpartum uterine disease. *PLoS One* 15(6)**,** e0228294. doi: 10.1371/journal.pone.0228294.

Johnson, T.J., Bielak, E.M., Fortini, D., Hansen, L.H., Hasman, H., Debroy, C., et al. (2012). Expansion of the IncX plasmid family for improved identification and typing of novel plasmids in drug-resistant Enterobacteriaceae. *Plasmid* 68(1)**,** 43-50. doi: 10.1016/j.plasmid.2012.03.001.

Jouini, A., Vinue, L., Slama, K.B., Saenz, Y., Klibi, N., Hammami, S., et al. (2007). Characterization of CTX-M and SHV extended-spectrum beta-lactamases and associated resistance genes in Escherichia coli strains of food samples in Tunisia. *J Antimicrob Chemother* 60(5)**,** 1137-1141. doi: 10.1093/jac/dkm316.

Liao, X.P., Xia, J., Yang, L., Li, L., Sun, J., Liu, Y.H., et al. (2015). Characterization of CTX-M-14-producing Escherichia coli from food-producing animals. *Front Microbiol* 6**,** 1136. doi: 10.3389/fmicb.2015.01136.

Lv, L., Partridge, S.R., He, L., Zeng, Z., He, D., Ye, J., et al. (2013). Genetic characterization of IncI2 plasmids carrying blaCTX-M-55 spreading in both pets and food animals in China. *Antimicrob Agents Chemother* 57(6)**,** 2824-2827. doi: 10.1128/AAC.02155-12.

Moyo, S.J., Maselle, S.Y., Matee, M.I., Langeland, N., and Mylvaganam, H. (2007). Identification of diarrheagenic Escherichia coli isolated from infants and children in Dar es Salaam, Tanzania. *BMC Infect Dis* 7**,** 92. doi: 10.1186/1471-2334-7-92.

Muller, D., Greune, L., Heusipp, G., Karch, H., Fruth, A., Tschape, H., et al. (2007). Identification of unconventional intestinal pathogenic Escherichia coli isolates expressing intermediate virulence factor profiles by using a novel single-step multiplex PCR. *Appl Environ Microbiol* 73(10)**,** 3380-3390. doi: 10.1128/AEM.02855-06.

Saenz, Y., Brinas, L., Dominguez, E., Ruiz, J., Zarazaga, M., Vila, J., et al. (2004). Mechanisms of resistance in multiple-antibiotic-resistant Escherichia coli strains of human, animal, and food origins. *Antimicrob Agents Chemother* 48(10)**,** 3996-4001. doi: 10.1128/AAC.48.10.3996-4001.2004.

Zhi, S., Banting, G., Li, Q., Edge, T.A., Topp, E., Sokurenko, M., et al. (2016). Evidence of Naturalized Stress-Tolerant Strains of Escherichia coli in Municipal Wastewater Treatment Plants. *Appl Environ Microbiol* 82(18)**,** 5505-5518. doi: 10.1128/AEM.00143-16.
