# Supplementary material for: Prevalence, Characteristics and Clonal Distribution of Extended-Spectrum β-Lactamase- and AmpC β-Lactamase-Producing Escherichia coli Following the Swine Production Stages, and Potential Risks to Humans
Source: Front Microbiol. 2021 Jul 21;12:710747. doi: 10.3389/fmicb.2021.710747 (PMC8334370; doi:10.3389/fmicb.2021.710747)
Supplement: Supplementary file 5 [file Table_2.pdf]

Supplementary Table 2. Comparison of the prevalence of plasmid-mediated antimicrobial resistance genes between ESBL/AmpC-EC and non-ESBL/AmpC-EC isolates.

Antibiotic classes	Antibiotic resistance gene	Prevalence (%) of.		OR (95% CI)	*p* - value
		ESBL/AmpC -EC (n = 161)	non-ESBL/AmpC -EC (n = 81)		
Phenicol	*catA*	4.4	0.0	7.9 (0.45-140.30)	0.16[†]
	cml	15.5	25.9	0.5 (0.10-2.89)	0.46
	floR	68.8	61.7	1.4 (0.54-3.46)	0.51
	catA, clmA, or floR	80.1	70.4	1.8 (0.48-6.53)	0.39
Tetracycline	*tetA*	52.8	56.8	0.9 (0.24-3.01)	0.80
	tetB	22.4	14.8	1.7 (0.60-4.60)	0.33
	tetD	3.7	0.0	6.8 (0.38-122.47)	0.19[†]
	tetA, tetB, or tetD	69.6	71.6	0.9 (0.28-3.14)	0.91
Quinolone	*qnrA*	0.0	0.0	-	-
	qnrB	0.6	2.5	0.3 (0.02-2.84)	0.26
	qnrC	0.0	0.0	-	-
	qnrS	14.3	19.8	0.7 (0.10-4.52)	0.69
	aac(6)-cr-Ib	0.0	0.0	-	-
	qnrA, qnrB, qnrC, qnrS, or aac(6)-cr-Ib	14.3	22.2	0.6 (0.09-3.99)	0.58
Aminoglycoside	*aac(3)-I*	0.0	0.0	-	-
	aac(3)-II	19.3	3.7	6.2 (1.25-30.70)	0.03*
	aac(3)-IV	8.1	1.2	7.0 (0.61-81.36)	0.12
	aac-(3)-I, II, or IV	23.6	4.9	5.9 (1.36-25.98)	0.02*
Sulfonamide/ Trimethoprim	*dfrIa*	1.2	7.3	0.2 (0.05-0.52)	< 0.01*
	dfrIb	0.0	0.0	-	-
	dfrII	0.0	0.0	-	-
	dfrVII	0.0	0.0	-	-

dfrXII	19.9	28.1	0.6 (0.17-2.26)	0.47
dfrIa, Ib, II, VII, or XII	21.1	32.9	0.5 (0.16-1.80)	0.31

CI, Confidence interval; OR, Odds ratio.

Statistically significant ($^{*}p < 0.05$, GEE; $^{\dagger}p < 0.05$, Chi-square test)
