# Supplementary material for: Prevalence, Characteristics and Clonal Distribution of Extended-Spectrum β-Lactamase- and AmpC β-Lactamase-Producing Escherichia coli Following the Swine Production Stages, and Potential Risks to Humans
Source: Front Microbiol. 2021 Jul 21;12:710747. doi: 10.3389/fmicb.2021.710747 (PMC8334370; doi:10.3389/fmicb.2021.710747)
Supplement: Supplementary file 6 [file Table_3.pdf]

Supplementary Table 3. Distributions of ST and phylogroup for each farm and production stage combination.

			Clone type<br>No. of isolate (%)					
Farm A	(n = 24)	Stage 1[a]<br>(n = 5)	ST48-A<br>3 (60.0)	ST5229-B1<br>2 (40.0)				
		Stage 2[b]<br>(n = 7)	ST641-B1<br>2 (28.6)	ST5229-B1<br>1 (14.3)	ST23-A<br>2 (28.6)	ST7203-A<br>1 (14.3)	ST205-B1<br>1 (14.3)	
		Stage 3[c]<br>(n = 9)	ST3944-A<br>2 (22.2)	ST75-B1<br>1 (11.1)	ST5229-B1<br>1 (11.1)	ST4014-B1<br>1 (11.1)	ST215-A<br>2 (22.2)	ST2628-B1<br>2 (22.2)
		Stage 4[d]<br>(n = 3)	ST75-B1<br>1 (33.3)	ST767-B1<br>1 (33.3)	ST10-A<br>1 (33.3)			
Farm B	(n = 23)	Stage 1<br>(n = 5)	ST101-B1<br>3 (60.0)	ST3076-B1<br>1 (20.0)	ST376-B1<br>1 (20.0)			
		Stage 2<br>(n = 8)	ST101-B1<br>6 (75.0)	ST3076-B1<br>1 (12.5)	ST641-B1<br>1 (12.5)			
		Stage 3<br>(n = 8)	ST101-B1<br>5 (62.5)	ST641-B1<br>1 (12.5)	ST953-A<br>1 (12.5)	ST218-A<br>1 (12.5)		
		Stage 4<br>(n = 2)	ST3076-B1<br>1 (50.0)	ST3944-A<br>1 (50.0)				
Farm C	(n = 15)	Stage 1<br>(n = 3)	ST75-B1<br>2 (66.7)	ST1642-B1<br>1 (33.3)				
		Stage 2<br>(n = 5)	ST75-B1<br>3 (60.0)	ST2628-B1<br>1 (20.0)	ST744-A<br>1 (20.0)			
		Stage 3<br>(n = 5)	ST75-B1<br>3 (60.0)	ST2628-B1<br>1 (20.0)	ST744-A<br>1 (20.0)			
		Stage 4<br>(n = 2)	ST2628-B1<br>2 (100.0)					
Farm D	(n = 15)	Stage 1<br>(n = 3)	ST457-F<br>1 (33.3)	ST5696-B1<br>1 (33.3)	ST1642-B1<br>1 (33.3)			
		Stage 2<br>(n = 7)	ST101-B1<br>1 (14.3)	ST457-F<br>6 (85.7)				
		Stage 3<br>(n = 4)	ST101-B1<br>2 (50.0)	ST10-A<br>2 (50.0)				
		Stage 4<br>(n = 1)	ST75-B1<br>1 (100.0)					

Farm		Stage	Clone type<br>No. of isolate (%)		
Farm E	(n = 18)	Stage 1<br>(n = 5)	ST101-B1<br>1 (20.0)	ST457-F<br>2 (40.0)	ST410-A<br>2 (40.0)
		Stage 2<br>(n = 7)	ST457-F<br>5 (71.4)	ST75-B1<br>1 (14.3)	ST12-B2<br>1 (14.3)
		Stage 3<br>(n = 3)	ST101-B1<br>1 (33.3)	ST457-F<br>1 (33.3)	ST3285-B1<br>1 (33.3)
		Stage 4<br>(n = 3)	ST101-B1<br>2 (66.7)	ST457-F<br>1 (33.3)	
Farm F	(n = 16)	Stage 1<br>(n = 6)	ST224-B1<br>2 (33.3)	ST457-F<br>3 (50.0)	ST1011-E<br>1 (16.7)
		Stage 2<br>(n = 5)	ST224-B1<br>3 (60.0)	ST457-F<br>2 (40.0)	
		Stage 3<br>(n = 3)	ST224-B1<br>3 (100.0)		
		Stage 4<br>(n = 2)	ST224-B1<br>1 (50.0)	ST457-F<br>1 (50.0)	
Farm G	(n = 15)	Stage 1<br>(n = 4)	ST75-B1<br>4 (100.0)		
		Stage 2<br>(n = 1)	ST101-B1<br>1 (100.0)		
		Stage 3<br>(n = 7)	ST101-B1<br>6 (85.7)	ST75-B1<br>1 (14.3)	
		Stage 4<br>(n = 3)	ST101-B1<br>3 (100.0)		
Farm H	(n = 6)	Stage 1<br>(n = 4)	ST224-B1<br>2 (50.0)	ST648-F<br>2 (50.0)	
		Stage 2<br>(n = 2)	ST648-F<br>2 (100.0)		
		Stage 3<br>(n = 0)			
		Stage 4<br>(n = 0)			
Farm I	(n = 4)	Stage 1<br>(n = 1)	ST617-A<br>1 (100.0)		
		Stage 2<br>(n = 2)	ST617-A<br>2 (100.0)		
		Stage 3<br>(n = 0)			
		Stage 4<br>(n = 1)	ST617-A<br>1 (100.0)		

Stage 1[a,] Weaning piglets; Stage 2[b], Growing pigs; Stage 3[c], Finishing pigs, Stage 4[d], Pregnant sows.
