# Supplementary material for: Prevalence, Characteristics and Clonal Distribution of Extended-Spectrum β-Lactamase- and AmpC β-Lactamase-Producing Escherichia coli Following the Swine Production Stages, and Potential Risks to Humans
Source: Front Microbiol. 2021 Jul 21;12:710747. doi: 10.3389/fmicb.2021.710747 (PMC8334370; doi:10.3389/fmicb.2021.710747)
Supplement: Supplementary file 7 [file Table_4.pdf]

Supplementary Table 4. Clonal population distribution of swine ESBL-EC isolates for each MLST sequence type.

CC – Phylogenetic group (No. of isolates)	ST - Phylogenetic group	No. of isolates in cluster 1	2	3	4	5	Total no.
CC101-B1 (37)	ST101-B1	10	19			2	31
	ST5229-B1		3			1	4
	ST5696-B1	1					1
	ST4014-B1					1	1
CC10-A (13)	ST10-A				3		3
	ST215-A				2		2
	ST48-A				3		3
	ST617-A				4		4
	ST218-A				1		1
CC23-A (4)	ST23-A				2		2
	ST410-A				2		2
CC648-F (4)	ST648-F			4			4
CC86-B1 (4)	ST641-B1		4				4
CC205-B1 (1)	ST205-B1					1	1
CC20-B1 (1)	ST376-B1	1					1
CC12-B2 (1)	ST12-B2			1			1
	ST457-F			22			22
	ST75-B1	15	1			1	17
	ST224-B1					11	11
	ST2628-B1	5				1	6
	ST3944-A				3		3
	ST3076-B1					3	3
	ST744-A				2		2
	ST1642-B1					2	2
	ST3285-B1	1					1
	ST1011-E					1	1
	ST7203-A				1		1
	ST953-A	1					1
	ST767-B1					1	1
	NT-B1		1				1
	NT-A				1		1
Total		34	28	27	24	25	138

CC, Clonal complex; ST, Sequence type; NT, non-typable.
